# Integrative Analysis of Deregulated miRNAs Reveals Candidate Molecular Mechanisms Linking H. pylori Infected Peptic Ulcer Disease with Periodontitis

**DOI:** 10.1155/2022/1498525

**Published:** 2022-01-29

**Authors:** Ning Li, Zhen Wang

**Affiliations:** ^1^Department of Prosthetic Dentistry, The Affiliated Stomatological Hospital of Wenzhou Medical University, Longyao Avenue No. 1288, Yongzhong Street, Longwan District, Wenzhou 325000, Zhejiang Province, China; ^2^Department of Stomatology, The Quzhou Affiliated Hospital of Wenzhou Medical University (Quzhou People's Hospital), Kecheng District, Minjiang Avenue No. 100, Quzhou 332400, Zhejiang Province, China

## Abstract

**Objective:**

Periodontitis is a highly prevalent oral infectious disease and has been increasingly associated with H. pylori infection, gastric inflammation, and gastric cancer but little is known about epigenetic machinery underlying this potentially bidirectional association. The present study is aimed at identifying key deregulated miRNA, their associated genes, signaling pathways, and compounds linking periodontitis with H. pylori-associated peptic ulcer disease.

**Methods:**

miRNA expression datasets for periodontitis-affected and H. pylori-associated peptic ulcer disease-affected tissues were sought from the GEO database. Differentially expressed miRNA (DEmiRNAs) were identified and the overlapping, shared-DEmiRNA between both datasets were determined. Shared-DEmiRNA-target networks construction and functional analyses were constructed using miRNet 2.0, including shared-DEmiRNA-gene, shared-DEmiRNA-transcription factor (TF), and shared-DEmiRNA-compound networks. Functional enrichment analysis for shared DEmiRNA-gene and shared DEmiRNA-TF networks was performed using the KEGG, Reactome, and Geno Ontology (GO) pathways.

**Results:**

11 shared-DEmiRNAs were identified, among which 9 showed similar expression patterns in both diseases, and 7 were overexpressed. miRNA hsa-hsa-mir-155-5p and hsa-mir-29a-3p were top miRNA nodes in both gene and TF networks. The topmost candidate miRNA-deregulated genes were PTEN, CCND1, MDM2, TNRC6A, and SCD while topmost deregulated TFs included STAT3, HIF1A, EZH2, CEBPA, and RUNX1. Curcumin, 5-fluorouracil, and the gallotanin 1,2,6-Tri-O-galloyl-beta-D-glucopyranose emerged as the most relevant linkage compound targets. Functional analyses revealed multiple cancer-associated pathways, PI3K pathways, kinase binding, and transcription factor binding among as enriched by the network-associated genes and TFs.

**Conclusion:**

Integrative analysis of deregulated miRNAs revealed candidate molecular mechanisms comprising of top miRNA, their gene, and TF targets linking H. pylori-infected peptic ulcer disease with periodontitis and highlighted compounds targeting both diseases. These findings provide basis for directing future experimental research.

## 1. Introduction

Periodontitis is a multifactorial infectious disease characterized by the inflammatory destruction of the supporting structures of the teeth in response to a dysbiotic dental plaque biofilm [1]. Periodontitis lesions represent chronic wounds that can impose significant systemic inflammatory burden and periodontal disease has been linked with a plethora of metabolic and other nonoral diseases [2]. Emerging evidence has highlighted an association of severe periodontal disease with peptic ulcer disease, chronic gastritis [3, 4], and also with gastric carcinogenesis [5]. Gastric inflammation and carcinogenesis are strongly associated with H. pylori infection [6–8]. Considering the serious global burden imposed by gastric cancer [9], the association of H. pylori infection and gastric inflammation with periodontal disease merits further mechanistic investigation. Several studies have identified the presence of a reservoir of H. pylori in the oral microflora of periodontitis patients [10–13]. In concurrence, the treatment of periodontitis has been found to improve gastric H. pylori elimination among peptic ulcer disease patients in multiple trials [14]. Others have suggested oral and gastric H. pylori infections may comprise a risk factor for periodontitis [13, 15]. Although it was earlier believed that oral detection of H. pylori did not represent viable bacteria but represented the genetic remnants derived from gastric colonization, viable H. pylori has been isolated from root canals [16]. In addition, live H. pylori bacterial could be cultured from saliva of gastric H. pylori-positive subjects [17]. Experimental data has shown that H. pylori infection and its virulence factor CagA can exert proinflammatory effects and promote the growth of periodontal pathogens [18].

Currently, very little is understood about the molecular mechanisms underlying the possibly syndemic association of periodontitis and H. pylori-associated gastric inflammatory conditions. MircoRNAs (miRNA) are small RNAs that play critical regulatory roles in controlling gene expression, thereby modulating cellular processes and playing key roles in mediating disease [19, 20]. The identification of disease-associated miRNA and their deregulation patterns can provide an insight into molecular pathogenesis events. In addition, such miRNA can serve as biomarkers or therapeutic drug target candidates [21]. Integrative bioinformatics has been applied successfully to decipher miRNA deregulation in oral diseases [22]. miRNAs have been identified as biomarkers of periodontal disease [23], and bioinformatic investigation has identified miR-125a-3p, miR-200a, and miR-142-3p as key deregulated miRNA in periodontitis [24]. In principle, deregulated miRNAs in periodontitis are not contratined to the tissue and can possibly enter circulation via exosomal carriage and thereby alter gene expression in remote sites [25]. In support, miR-146a has been implicated in the association of periodontitis with cardiovascular disease [26].

With this context, the present study is aimed at performing an integrated analysis of miRNA deregulation patterns in periodontitis and H. pylori-associated peptic ulcer disease to identify candidate linkage miRNAs, their associated genes, signaling pathways, and relevant compounds. The broader goal of this approach was to advance the understanding of molecular linkage mechanisms in context of these diseases and provide theoretical basis for driving future experimental research.

## 2. Materials and Methods

### 2.1. Microarray Data

miRNA expression datasets of the target conditions were sought from the GEO database (https://www.ncbi.nlm.nih.gov/geo/). The miRNA expression dataset for periodontitis, GSE54710 [27] (acquired on the Agilent-031181 Unrestricted_Human_miRNA_V16.0_Microarray 030840 platform), and the dataset GSE32174, for H. pylori-associated duodenal ulcers [28] (acquired on the Illumina Human v1 microRNA expression beadchip) were identified for analysis. The GSE54710 dataset contains 200 samples, with 158 periodontitis-affected and 41 control tissue samples. The GSE32174 dataset contains samples from antral biopsies, including a total of 48 samples with 9 H. pylori-negative and 39 H. pylori-positive samples identified in the metadata.

### 2.2. Identification of DEmiRNA and Shared-DEmiRNA

The microarray data expression matrix and metadata were downloaded using the “GEOquery” v 2.32.0 package in R (https://www.r-project.org/). Next, differentially expressed miRNA was identified using the R package “limma” v.3.22.7 [29]. miRNA with expression levels above the median and in more than 2 samples was considered as “expressed” and utilized in the analysis. The assigned labels of the samples (periodontitis affected versus controls for GSE54710, and H. pylori-positive versus negative for GSE32174) were utilized for grouping, and DEmiRNA was identified using an FDR adjusted *p* value < 0.05. Probe IDs were mapped to miRBase miRNA IDs (version 8.). The two DEmiRNA lists were processed using the R package “Venndiagram” v 1.6.9 [30], and the shared-DEmiRNA was obtained. These were considered the shared DEmiRNA and were subjected to further analysis.

### 2.3. Shared-DEmiRNA-Target Network Construction and Functional Enrichment Analyses

DEmiRNA target networks were constructed using miRNet 2.0 (https://www.mirnet.ca/) [31]. For the shared-DEmiRNA-gene network, target genes were selected from the 3 databases (miRTarBase v8.0, TarBase v8.0, and miRecords). A “minimum network” was selected in order to reduce network complexity and retain the key features demonstrating network connectivity. It was computed using all the seed or query nodes. To construct a “minimum network,” pair-wise shortest paths between the seed nodes are determined, and any nodes not on the shortest paths are removed. A similar approach was used to construct a shared-DEmiRNA-transcription factor (TF) network, which utilizes TransmiR v2.0., and a shared-DEmiRNA-small molecule network, which is based upon data from SM2miR and PharmacomiR, each. Functional enrichment analysis was conducted with a hypergeometric test algorithm for shared DEmiRNA-gene and shared DEmiRNA-TF networks using the KEGG, Reactome, and Geno Ontology (GO) pathways with the query set as “all genes” in the 2 minimum networks and top 10 significantly enriched functions ranked by *p* value were tabulated.

## 3. Results

### 3.1. Identification of DEmiRNA and Shared-DEmiRNA

**Figure 1:**
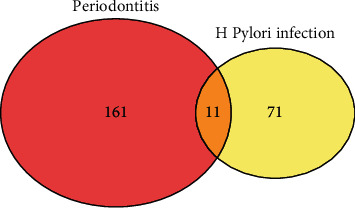
Venn diagram depicting the shared DEmiRNAs between periodontits-affected and H. pylori-infected tissues identified using the GEO datasets GSE54710 and GSE32174, respectively.

After filtering, 1200 miRNA were retained in the analysis for the GSE54710 dataset, and a total of 172 significant periodontitis-associated DEmiRNA were identified, whereas 425 miRNAs were retained for the GSE32174 dataset analysis and 82 significant H. pylori infection-associated DEmiRNA were identified (Supplementary Table-S1). 177 DEmiRNA were overexpressed, and 55 DEmiRNA were underexpressed as compared to controls in periodontitis-affected tissue. Similarly, 49 miRNAs were overexpressed in H. pylori-infected tissue, while 33 were underexpressed. The overlap between the two DEmiRNA lists was sought using Venn diagram, and a total of the 11 shared-DEmiRNA were identified (Figure 1).

**Figure 2:**
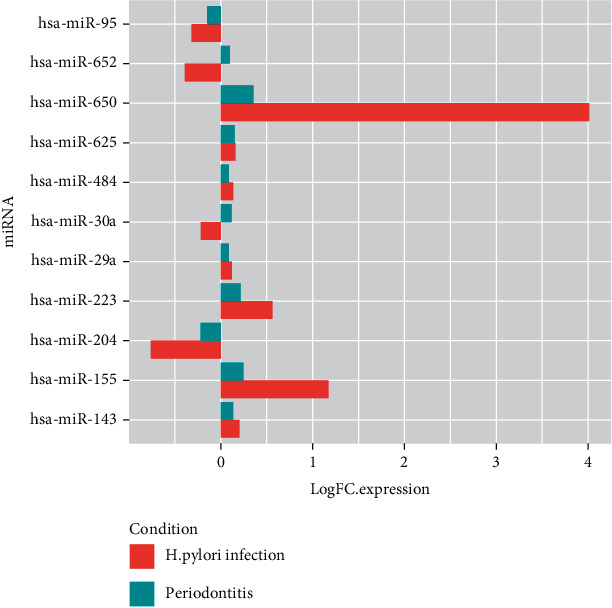
Bar plots depicting the log FC expression values of the shared-DEmiRNAs in periodontitis and H. pylori infection.

Corresponding expression patterns in the 2 conditions are depicted in Figure 2. Similar expression patterns were evident in case of 9 shared-DE miRNAs, where overexpression was noted in 7 and underexpression was seen in 2 shared-DEmiRNAs as compared to control tissues in both conditions. 2 shared-DEmiRNAs, hsa-miR-652 and has-miR-30a, showed opposing expression patterns in both the conditions, with underexpression as compared to control in case of H. pylori infection but overexpression in case of periodontitis-affected tissue.

### 3.2. Shared-DEmiRNA-Target Networks and Functional Analyses

**Figure 3:**
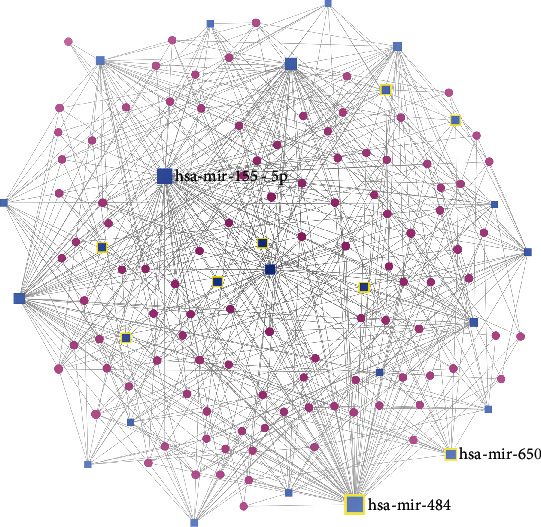
Shared DEmiRNA-genes minimum network visualized using the visual analytics platform “miRnet.” The seed nodes are highlighted. It comprised of 105 genes and 27miRNA with 524 edges.

Shared-DEmiRNA-gene network: the shared-DEmiRNA-gene minimum network comprised of 105 genes and 27miRNA with 524 edges (Figure 3, Supplementary Table S2). The top DEmiRNA nodes with the highest degrees in the network were hsa-mir-484, hsa-mir-155-5p, hsa-mir-29a-3p, and hsa-mir-30a-5p. The top 5 gene nodes with the highest degrees in the network included PTEN, CCND1, MDM2, and TNRC6A. The top 10 enriched functions in each are listed in Table 1(a). The reactome analysis showed signaling by SCF-KIT, oncogene-induced senescence, pre-NOTCH transcription and translation, and multiple PI3K signaling-related pathways among the top enriched pathways. GO biological process analysis showed negative regulation of multiple processes such as cellular and RNA metabolic processes and transcription. Top enriched GO molecular functions included multiple binding associated functions including protein kinase, enzyme, TF, and chromatin binding. Top GO cellular components enriched included cytosol, nucleoplasm, and nuclear lumen.

**Figure 4:**
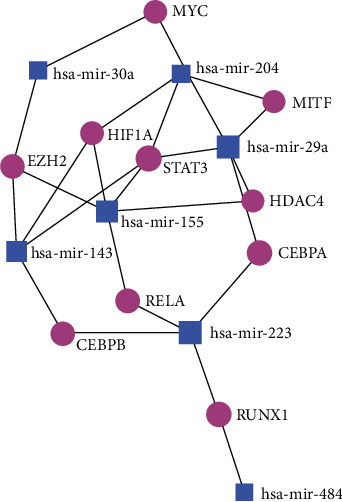
Shared-DEmiRNA-TF minimum network comprised of 10 TFs and 7 miRNA with 24 edges.

Shared-DEmiRNA-TF network: the shared-DEmiRNA-TF minimum network comprised of 10 TFs and 7miRNA with 24 edges (Figure 4, Supplementary Table S3). The top 5 TFs included STAT3, HIF1A, EZH2, CEBPA, and RUNX1, while hsa-mir-29a and hsa-mir-155 were the topmost miRNA nodes. The top enriched KEGG, Reactome, and GO pathways are listed in Table 1(b). These included the reactome pathways of responses to stress, cellular senescence, and multiple NOTCH signaling-related pathways. GO BP pathway analysis showed the regulation of cell development, differentiation, and transcription as among those implicated. GO molecular functions enhanced among the TFs in the network included multiple transcription-related functions.

**Figure 5:**
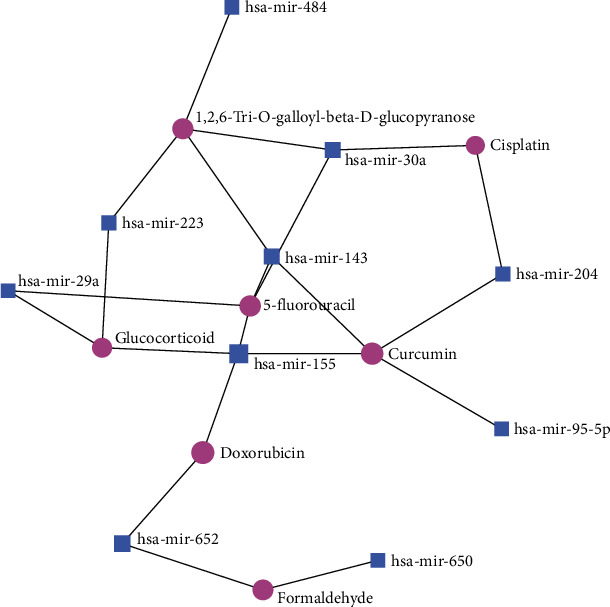
The shared DE-miRNA-small molecule minimum network comprised of 7 compounds and 10 miRNAs with 21 edges.

Shared DE-miRNA-small molecule network: the shared DE-miRNA-small molecule minimum network comprised of 7 compounds and 10 miRNAs with 21 edges (Figure 5, Supplementary Table S4). The 7 compounds in this network included 5-fluorouracil, curcumin, 1,2,6-Tri-O-galloyl-beta-D-glucopyranose, glucocorticoid, cisplatin, doxorubicin, and formaldehyde. The compound with the highest degree and betweeness was curcumin, followed by 5-fluorouracil.

## 4. Discussion

The present bioinformatics study explored miRNA-mediated regulatory mechanisms implicated in the association of gastric H. pylori infection with periodontitis by identifying common deregulated miRNA in both conditions. Network analysis was applied to identify the key candidate genes and TFs that may act as linkage molecular mechanisms operating via miRNA-mediated deregulation. In addition, small molecules and compounds associated with the shared DEmiRNA were identified. Similar expression patterns of the shared DEmiRNA (Figure 1) were evident for most of the deregulated miRNA in both conditions, suggesting common deregulatory patterns in host immune mechanisms implicated in the two conditions. The shared DEmiRNA with the highest degrees included hsa-mir-484 and hsa-mir-155, followed by hsa-mir-29a, which were found overexpressed in both conditions, and hsa-mir-30a which showed contradictory expression patterns. Both miR 155 [32–34] and miR-484 [35, 36] have been widely implicated in inflammation and cancer. miRNA 29a is shown to be an important regulator of the intestinal barrier function [37] and also implicated as a cancer biomarker [38, 39]. miRNA is an important effector of the host-bacterial interaction, whereby pathogenic microbiota alter host miRNA production to enhance their survival [40]. Chronic gastric H. pylori infection is an important risk factor for gastric cancer [6–8], while severe chronic periodontitis has also been linked to gastric cancer [5, 41]. H. pylori infection has been associated with higher levels of key periodontal pathogens and was found to enhance the expression of IL-8 and Wnt5a, suggesting it may act as an aggravating factor for periodontal disease [18]. Overall, a bidirectional and syndemic link between H. pylori and periodontitis is likely, and the key deregulated miRNA identified in this study may contribute to the oncogenic risks for oral and gastric carcinogenesis arising from both conditions.

miRNA-mediated deregulation of gene expression is likely to be a key molecular mechanism linking periodontitis with systemic diseases [25]. The genes with the highest degrees in shared-DEminRNA-gene network included PTEN, CCND1, MDM2, SCD, and TNRC6A, and these may be important miRNA-deregulated mediators linking H. pylori infection and periodontitis. PTEN (Phosphatase and Tensin Homolog) signaling is involved in regulating multiple cellular processes, and its deregulation has been associated with cancer [42]. PTEN is a well-known tumor suppressor and is commonly inactivated in multiple cancers, playing key role in regulating the PI3K/AKT/mTOR pathway which effects cancer cell survival [43]. Multiple studies have associated the deregulation of PTEN with gastric carcinogenesis, leading to inhibition of its tumor suppressor function [44–46]. miRNA-mediated alteration of PTEN signaling has been shown as an important pathway in gastric cancer development [47], and its phosphorylation and inhibition by H. pylori infection have been demonstrated [48]. PTEN downregulation is also documented in periodontitis [49] and P. ginivalis, and the keystone periodontal pathogen was found to promote esophageal carcinoma cell by modulation of PTN signaling [50]. CCND1 has been associated with gastric cancer [51–53], and its polymorphisms have been hypothesized to mediate risk for periodontitis-linked oral cancer [54], suggesting similar phenomena in gastric cancer. MDM2 has been implicated in oxidative stress-mediated P53 gene upregulation and damage of gingival fibroblasts [55], and its upregulation is also associated with gastric H. pylori infection [56]. SCD or stearoyl CoA desaturase-1 is implicated in fatty acid synthesis and has been associated with metabolic disorders [57] and gastric cancer [58]. In a rat model of periodontitis, SCD-1 was found markedly upregulated in the liver and associated with metabolic aberration [59]. miRNA-mediated deregulation of TNRC6A has been implicated in gastric and colorectal cancers [60] although knowledge about its role in periodontitis remains scarce.

In the shared-DEmiRNA TF network, STAT3, EZH2, HIF1A, CEBPA, and RUNX1 showed the highest degrees. STAT3 (signal transducer and activator of transcription 3) activation is implicated in inflammation-associated carcinogenesis, and its phosphorylation by H. pylori has been documented [61]. STAT3 pathway activation is documented in periodontitis [62] and is also found to contribute to neuroinflammation [63]. EZH2 encodes for a methyltransferase enzyme that is involved in osteoclast regulation and is proposed as a target for periodontitis [64]. EZH2 was found downregulated in response to short-chain fatty acids produced by periodontal pathogens, which contributes to cancer development [65]. Its expression was, however, reported to be upregulated by H. pylori virulence factor cytotoxin-associated gene A [66] and increasing expression levels associated with the multistep gastric carcinogenesis process [67]. HIF1A, involved in cellular responses to oxidative stress, has been implicated in the immune-inflammatory response in periodontitis [68] and is also upregulated in H. pylori-mediated gastric inflammation [69] and carcinogenesis [70]. Consistent with our finding, CEBPA, an NF-*κ*B-related associated gene, has been previously reported in a bioinformatics study as a key TF involved in H. pylori-mediated immune deregulation [71, 72] and validated as downregulated in infected gastric tissues [73]. In periodontitis, lower serum levels of CEBPA have been reported [74]. The RUNX genes are important regulators of cellular development, and reduced RUX1 expression in gastric cancer cell lines is documented [75]. RUNX1 is involved in alveolar osteocalstogenesis [76].

Functional enrichment analysis of the shared-DEmiRNA gene and TF networks showed several cancer-related KEGG pathways as enriched, supporting the emerging findings of increased gastric and esophageal carcinogenesis risk in periodontitis, evidenced in large sample prospective data [77]. Reactome analysis indicated multiple PI3K pathway alterations as shared linkages, which are implicated in H. pylori-mediated inflammation [78] and widely understood to function in carcinogenesis [79]. Multiple GO molecular functions related to kinase binding were enriched, which are pivotal to the regulation of innate immune responses and inflammation [80].

In the present study, small compounds targeted by the shared-DEmiRNA were also analyzed. 5-fluorouracil, curcumin, and 1,2,6-Tri-O-galloyl-beta-D-glucopyranose were identified as the top candidates. H. pylori CagA protein is found to reduce the sensitivity of gastric cancer cells to the antineoplastic agent 5-fluorouracil [81], which is also shown to exacerbate periodontitis progression [82]. However, the role of periodontitis in modulating resistance to 5-fluorouracil in gastric cancer remains unknown. Curcumin, a phytochemical, has shown anti-inflammatory effects in periodontitis [83] and also shows anti-infective efficacy against gastric H. pylori [84]. Taken together, these findings suggest curcumin might be a candidate therapeutic for cotreatment of both these conditions. 1,2,6-Tri-O-galloyl-*β*-d-glucopyranose (1,2,6-TGGP) is a gallotanin that has shown efficacy against multiple bacteria and diseases [85] and can be considered a viable candidate phytochemical to combat both H. pylori gastric infections and periodontitis. Future studies are warranted to investigate the identified molecular candidates in the context of the association of these diseases.

The major limitation of the present study is the lack of validating experimental data to verify to candidate molecular linkage mechanisms discovered through bioinformatics. The datasets used in the present study include small sample numbers and are from single centers, which can limit the robustness of the predictions. Similar studies using multiple large datasets with comprehensive metadata are essential. An important caveat is that deregulated exosomal or circulatory miRNA was not analyzed in the present investigation, and these may be more important in the pathogenic mechanisms linking periodontitis with systemic disease [25]. In addition, future studies may address deregulated, genes, or other noncoding RNAs as linkage mechanisms. Future studies should be designed to verify the miRNAs, their associated genes, TF, and functional pathways that constitute putative linkages between H. pylori-infected gastric diseases and periodontitis using validation experiments using clinical cohorts, animal, and cell models. Such data can permit the discovery of molecular targets for both conditions. Furthermore, as the association is likely to be bidirectional in nature, a comprehensive understanding the biological mechanisms involved via both experimental and clinical research is warranted.

## 5. Conclusions

Integrative analysis of common deregulated miRNAs in H. pylori-associated peptic ulcer and periodontitis-affected tissues revealed key candidate linkage molecular mechanisms comprising of miRNAs (including hsa-mir-155-5p, hsa-mir-484, and hsa-mir-29a-3p), genes (including PTEN, CCND1, MDM2, TNRC6A, and SCD), and TF targets (including STAT3, HIF1A, EZH2, CEBPA, and RUNX1) and highlighted the most relevant compounds (including curcumin, 5-fluorouracil, and 1,2,6-tri-O-galloyl-beta-D-glucopyranose) in this context. These findings provide a theoretical basis for directing future experimental studies.

## Figures and Tables

**Table 1a:** (a) Top enriched pathways in the shared DEmiRNA-gene network

KEGG pathway	*p* value
Glioma	4.48E-06
Neurotrophin signaling pathway	4.51E-06
Prostate cancer	3.14E-05
Pathways in cancer	7.11E-05
Melanoma	7.23E-05
Chronic myeloid leukemia	0.000108
Bladder cancer	0.000225
Cell cycle	3.00E-04
Cysteine and methionine metabolism	0.000422
p53 signaling pathway	0.000713
Reactome pathway	
Signaling by SCF-KIT	1.43E-05
Oncogene induced senescence	1.70E-05
Pre-NOTCH transcription and translation	4.81E-05
PI3K events in ERBB4 signaling	7.45E-05
PIP3 activates AKT signaling	7.45E-05
PI3K events in ERBB2 signaling	7.45E-05
PI-3K cascade:FGFR1	7.45E-05
PI-3K cascade:FGFR2	7.45E-05
PI-3K cascade:FGFR3	7.45E-05
PI-3K cascade:FGFR4	7.45E-05
GO-BP	
Negative regulation of metabolic process	1.46E-09
Negative regulation of cellular metabolic process	1.96E-09
Negative regulation of transcription from RNA polymerase II promoter	2.02E-09
Negative regulation of cellular biosynthetic process	5.27E-09
Negative regulation of biosynthetic process	7.61E-09
Negative regulation of transcription, DNA-dependent	9.74E-08
Negative regulation of transcription, DNA-dependent	9.74E-08
Negative regulation of RNA metabolic process	1.93E-07
Negative regulation of nucleobase-containing compound metabolic process	3.08E-07
Generation of neurons	1.23E-06
GO-MF	
Protein kinase binding	4.93E-09
Kinase binding	2.20E-08
Negative regulation of transcription, DNA-dependent	3.35E-08
Chromatin binding	4.92E-07
Transcription factor binding	1.78E-06
Enzyme binding	4.41E-06
Transcription from RNA polymerase II promoter	8.05E-06
Transcription corepressor activity	8.85E-05
SMAD binding	1.00E-04
Protein binding transcription factor activity	0.000788
GO-CC	
Cytosol	4.70E-07
Nucleoplasm	2.96E-06
Nuclear lumen	5.22E-06
Organelle lumen	8.75E-06
Nucleoplasm part	9.20E-06
Macromolecular complex	1.19E-05
Membrane-enclosed lumen	1.35E-05
Transcription factor complex	1.65E-05
Membrane-bounded vesicle	3.32E-05
Nuclear chromatin	5.44E-05

**Table 1b:** (b) Top enriched pathways in shared DEmiRNA-TF network

KEGG pathway	*p* value
Acute myeloid leukemia	2.72E-08
Pathways in cancer	5.71E-06
Chronic myeloid leukemia	0.000286
Epstein-Barr virus infection	0.000549
Transcriptional misregulation in cancer	0.000553
Adipocytokine signaling pathway	0.00604
Pancreatic cancer	0.00721
Small cell lung cancer	0.00961
Toxoplasmosis	0.0128
Jak-STAT signaling pathway	0.0145
Reactome pathway	
Cellular responses to stress	6.47E-05
Cellular senescence	0.000306
NOTCH1 intracellular domain regulates transcription	0.000706
Transcriptional regulation of white adipocyte differentiation	0.00143
Signaling by NOTCH1	0.00159
Senescence-associated secretory phenotype (SASP)	0.00228
Signaling by NOTCH	0.00323
DEx/H-box helicases activate type I IFN and inflammatory cytokines production	0.00724
Binding of TCF/LEF:CTNNB1 to target gene promoters	0.00724
IkBA variant leads to EDA-ID	0.00724
GO-BP	
Regulation of cell differentiation	3.46E-11
Regulation of developmental process	1.51E-09
Cell proliferation	1.72E-09
Transcription from RNA polymerase II promoter	1.97E-09
Positive regulation of transcription from RNA polymerase II promoter	3.80E-09
Regulation of multicellular organismal process	2.44E-08
Regulation of transcription from RNA polymerase II promoter	2.55E-08
Positive regulation of transcription, DNA-dependent	1.35E-07
Positive regulation of transcription, DNA-dependent	1.35E-07
Positive regulation of RNA metabolic process	2.13E-07
GO-MF	
Transcription factor binding	9.34E-11
Transcription from RNA polymerase II promoter	1.72E-09
RNA polymerase II distal enhancer sequence-specific DNA binding transcription factor activity	5.64E-09
DNA binding	6.20E-08
Sequence-specific DNA binding	8.61E-08
Positive regulation of transcription, DNA-dependent	1.22E-07
Negative regulation of transcription, DNA-dependent	6.69E-07
Protein dimerization activity	7.12E-07
Protein heterodimerization activity	8.13E-05
Protein kinase binding	8.30E-05
GO-CC	
Nuclear lumen	1.51E-05
Nucleoplasm	1.65E-05
Nuclear part	7.71E-05
Organelle lumen	8.69E-05
Nucleoplasm part	9.60E-05
Membrane-enclosed lumen	9.90E-05
Nucleus	0.000389
Transcription factor complex	0.000649
Nuclear matrix	0.00118
Protein complex	0.00294

## Data Availability

The datasets utilized in the present study are publically available.

## Supplementary Materials

Supplementary Materials Table S1a: significant DEmiRNA in periodontitis (GSE54710). Table S1b: significant DEmiRNA in H. pylori-infected gastric tissue (GSE32174). Table S2a: shared-DEmiRNA-gene network interactions. Table S2b: KEGG pathway enrichment analysis of shared-DEmiRNA-gene network. Table S2c: reactome pathway enrichment analysis of shared-DEmiRNA-gene network. Table S2d: GO–biological pathways enrichment analysis of shared-DEmiRNA-gene network. Table S2e: GO-molecular functions enrichment analysis of shared-DEmiRNA-gene network. Table S2f: GO cellular component enrichment analysis of shared-DEmiRNA-gene network. Table S3a: shared-DEmiRNA-TF network interactions. Table S3b: KEGG pathway enrichment analysis of shared-DEmiRNA-TF network interactions. Table S3c: reactome pathway enrichment analysis of shared-DEmiRNA-TF network interactions. Table S3d: GO-BP pathway enrichment analysis in shared-DEmiRNA-TF network interactions. Table S3e: GO-molecular functions enrichment analysis in shared-DEmiRNA-TF network interactions. Table S3f: GO cellular component enrichment analysis in shared-DEmiRNA-TF network interactions. Table S4: shared-DEmiRNA-compound network interactions.
