# Supplementary material for: Integrative Analysis of Deregulated miRNAs Reveals Candidate Molecular Mechanisms Linking H. pylori Infected Peptic Ulcer Disease with Periodontitis
Source: Dis Markers. 2022 Jan 29;2022:1498525. doi: 10.1155/2022/1498525 (PMC8817886; doi:10.1155/2022/1498525)
Supplement: Supplementary Materials — Table S1a: significant DEmiRNA in periodontitis (GSE54710). Table S1b: significant DEmiRNA in H. pylori-infected gastric tissue (GSE32174). Table S2a: shared-DEmiRNA-gene network interactions. Table S2b: KEGG pathway enrichment analysis of shared-DEmiRNA-gene network. Table S2c: reactome pathway enrichment analysis of shared-DEmiRNA-gene network. Table S2d: GO–biological pathways enrichment analysis of shared-DEmiRNA-gene network. Table S2e: GO-molecular functions enrichment analysis of shared-DEmiRNA-gene network. Table S2f: GO cellular component enrichment analysis of shared-DEmiRNA-gene network. Table S3a: shared-DEmiRNA-TF network interactions. Table S3b: KEGG pathway enrichment analysis of shared-DEmiRNA-TF network interactions. Table S3c: reactome pathway enrichment analysis of shared-DEmiRNA-TF network interactions. Table S3d: GO-BP pathway enrichment analysis in shared-DEmiRNA-TF network interactions. Table S3e: GO-molecular functions enrichment analysis in shared-DEmiRNA-TF network interactions. Table S3f: GO cellular component enrichment analysis in shared-DEmiRNA-TF network interactions. Table S4: shared-DEmiRNA-compound network interactions. [file 1498525.f1.docx]

**Supplementary Materials**

**Table S1a: Significant DEmiRNA in Periodontitis (GSE54710)**

| **miRNA** | **logFC** | **AveExpr** | **t** | **P Value** | **Adj P Value** | **B** |
| --- | --- | --- | --- | --- | --- | --- |
| hsa-miR-3917 | 0.441079601 | 1.975532843 | 9.789827429 | 0.00 | 1.20E-15 | 31.90047558 |
| hsa-miR-139-3p | 0.19297076 | 1.821132065 | 8.519244514 | 0.00 | 2.36E-12 | 23.75703121 |
| hsa-miR-650 | 0.35435861 | 1.636234215 | 7.89489995 | 0.00 | 7.63E-11 | 19.94376799 |
| hsa-miR-33a | -0.287690667 | 1.611440406 | -6.642373377 | 0.00 | 8.63E-08 | 12.78102752 |
| hsa-miR-145 | 0.230837915 | 2.73518619 | 6.167885498 | 0.00 | 9.09E-07 | 10.26907244 |
| hsa-miR-140-3p | 0.229148541 | 2.738580201 | 6.117174902 | 0.00 | 9.91E-07 | 10.00795939 |
| hsa-miR-486-5p | 0.298490733 | 2.788043707 | 6.041644241 | 0.00 | 1.26E-06 | 9.621776025 |
| hsa-miR-219-5p | -0.154768905 | 1.447753631 | -5.892283452 | 0.00 | 2.40E-06 | 8.867881965 |
| hsa-miR-375 | -0.177815677 | 1.478115927 | -5.835882522 | 0.00 | 2.57E-06 | 8.586634625 |
| hsv1-miR-H8 | 0.229237703 | 1.714038272 | 5.848548656 | 0.00 | 2.57E-06 | 8.649629426 |
| hsa-miR-125b-2* | -0.139994161 | 1.582789964 | -5.69762532 | 0.00 | 4.71E-06 | 7.905330211 |
| hsa-miR-3659 | -0.078750238 | 1.335868692 | -5.659825885 | 0.00 | 5.07E-06 | 7.721097882 |
| hsa-miR-4259 | 0.135046702 | 1.483877985 | 5.649751653 | 0.00 | 5.07E-06 | 7.672145734 |
| hsa-miR-3679-5p | 0.213142194 | 2.78575195 | 5.575582656 | 0.00 | 6.82E-06 | 7.313692634 |
| hcmv-miR-UL70-3p | 0.277852029 | 2.697032241 | 5.534706697 | 0.00 | 7.80E-06 | 7.11761608 |
| hsa-miR-141* | -0.045266242 | 1.292843415 | -5.337114404 | 0.00 | 1.62E-05 | 6.184813749 |
| hsa-miR-202 | 0.252252781 | 1.718177189 | 5.371617866 | 0.00 | 1.62E-05 | 6.345885509 |
| hsa-miR-483-5p | 0.301261469 | 2.487434905 | 5.356891536 | 0.00 | 1.62E-05 | 6.277044139 |
| hsa-miR-671-5p | 0.291678273 | 2.721707419 | 5.345614953 | 0.00 | 1.62E-05 | 6.224424716 |
| hsa-miR-1246 | -0.264726923 | 2.760268618 | -5.27065613 | 0.00 | 1.62E-05 | 5.876761125 |
| hsa-miR-1260 | -0.198295541 | 2.94203384 | -5.313959371 | 0.00 | 1.62E-05 | 6.077154996 |
| hsa-miR-1260b | -0.185053916 | 2.945057291 | -5.275071862 | 0.00 | 1.62E-05 | 5.897139272 |
| hsa-miR-205* | -0.271802559 | 2.06179901 | -5.281763837 | 0.00 | 1.62E-05 | 5.928046538 |
| hsa-miR-2117 | 0.070274643 | 1.330544978 | 5.300060056 | 0.00 | 1.62E-05 | 6.012699059 |
| hsa-miR-23b* | -0.056163665 | 1.356868236 | -5.304700809 | 0.00 | 1.62E-05 | 6.034205749 |
| hsv1-miR-H17 | 0.258116296 | 2.217639887 | 5.293687879 | 0.00 | 1.62E-05 | 5.983191449 |
| hsa-miR-720 | -0.080047331 | 3.717887438 | -5.250413382 | 0.00 | 1.72E-05 | 5.783507639 |
| hsa-miR-663 | 0.219687787 | 2.447605379 | 5.084475142 | 0.00 | 3.63E-05 | 5.029365697 |
| hsa-miR-3652 | 0.275897291 | 2.351688718 | 5.07655471 | 0.00 | 3.64E-05 | 4.993833062 |
| hsa-miR-210 | -0.175661152 | 2.849758748 | -5.051971521 | 0.00 | 3.85E-05 | 4.883819616 |
| hsa-miR-575 | 0.173208287 | 3.036249917 | 5.049979404 | 0.00 | 3.85E-05 | 4.874922621 |
| hsa-miR-617 | -0.140418036 | 1.401967493 | -4.923324409 | 0.00 | 6.68E-05 | 4.314859783 |
| hsa-miR-708* | -0.017500255 | 1.247226743 | -4.894237724 | 0.00 | 7.39E-05 | 4.187806248 |
| hsa-miR-645 | 0.074302313 | 1.388364405 | 4.816543456 | 0.00 | 0.000101887 | 3.851331562 |
| hsa-miR-155 | 0.24569401 | 2.34142817 | 4.74099881 | 0.00 | 0.000138629 | 3.528250657 |
| hsa-miR-4303 | 0.031530367 | 1.277068265 | 4.674354635 | 0.00 | 0.000177622 | 3.246612455 |
| hsa-miR-659 | 0.096160782 | 1.418302165 | 4.67221028 | 0.00 | 0.000177622 | 3.237603355 |
| hsa-miR-451 | 0.169977804 | 3.672951662 | 4.631273883 | 0.00 | 0.000206858 | 3.066253016 |
| hsa-miR-3926 | 0.109540429 | 1.530534038 | 4.604677626 | 0.00 | 0.000226278 | 2.955577187 |
| hsa-miR-424* | 0.139120026 | 1.449641049 | 4.597122904 | 0.00 | 0.00022697 | 2.924233177 |
| hsa-miR-623 | 0.111397541 | 1.498689403 | 4.592442654 | 0.00 | 0.00022697 | 2.904835978 |
| hsa-miR-3663-5p | 0.041667701 | 1.294014866 | 4.58467444 | 0.00 | 0.000229146 | 2.872675986 |
| hsa-miR-1273d | 0.045222623 | 1.301205131 | 4.571635413 | 0.00 | 0.000236795 | 2.81879401 |
| hsa-miR-223 | 0.215341736 | 3.10770367 | 4.557659829 | 0.00 | 0.000240331 | 2.761179677 |
| hsa-miR-4306 | 0.215542737 | 2.54533525 | 4.561110092 | 0.00 | 0.000240331 | 2.775390109 |
| hsa-miR-19b-1* | -0.024623848 | 1.266138986 | -4.517238594 | 0.00 | 0.000279679 | 2.595348388 |
| hsa-miR-211 | -0.208317819 | 1.522488629 | -4.450181291 | 0.00 | 0.00036417 | 2.322894262 |
| hsa-miR-510 | -0.033863533 | 1.266685059 | -4.424203144 | 0.00 | 0.000397947 | 2.218240122 |
| hsa-miR-514 | -0.018590943 | 1.246754323 | -4.396667751 | 0.00 | 0.000437691 | 2.107861226 |
| hsa-miR-3121-3p | 0.025473283 | 1.264037906 | 4.372247752 | 0.00 | 0.000475121 | 2.010444794 |
| hsa-miR-24-1* | -0.180219644 | 1.612006216 | -4.329090376 | 0.00 | 0.000557498 | 1.83937526 |
| hsa-miR-3156-5p | -0.22117266 | 2.212188868 | -4.314891369 | 0.00 | 0.000579909 | 1.78339909 |
| hsa-miR-1972 | 0.091837666 | 1.588994637 | 4.258667711 | 0.00 | 0.000717202 | 1.56324879 |
| hsv1-miR-H15 | -0.074327397 | 1.369779681 | -4.206081756 | 0.00 | 0.000872331 | 1.359518393 |
| hsa-miR-432 | 0.076797926 | 1.486870383 | 4.155466873 | 0.00 | 0.001050895 | 1.165424228 |
| hsa-miR-564 | 0.109569672 | 1.623014992 | 4.118395659 | 0.00 | 0.001197555 | 1.024518085 |
| hsa-miR-200c* | -0.019171824 | 1.292309947 | -4.037696885 | 0.00 | 0.001603853 | 0.721470534 |
| hsa-miR-765 | 0.233208939 | 2.007760401 | 4.035899096 | 0.00 | 0.001603853 | 0.714777096 |
| hsa-miR-493 | 0.032921221 | 1.312634816 | 4.002101034 | 0.00 | 0.001800261 | 0.589412795 |
| hsa-miR-3141 | 0.216663366 | 2.381832626 | 3.990815766 | 0.00 | 0.001842629 | 0.547752835 |
| hsa-miR-584 | 0.102635936 | 1.541423755 | 3.985106506 | 0.00 | 0.001842629 | 0.526715032 |
| hsa-miR-936 | 0.087189264 | 1.381049345 | 3.983440773 | 0.00 | 0.001842629 | 0.520581879 |
| hsa-miR-186 | 0.153763309 | 2.021753601 | 3.972741889 | 0.00 | 0.001884995 | 0.48124112 |
| hsa-miR-3609 | 0.058077823 | 1.38345608 | 3.969444163 | 0.00 | 0.001884995 | 0.46913326 |
| hsa-miR-4271 | 0.189001271 | 2.385993497 | 3.965271714 | 0.00 | 0.001886332 | 0.453826072 |
| hsa-miR-125a-3p | 0.195258973 | 2.376725422 | 3.944035316 | 0.00 | 0.002017149 | 0.376130372 |
| hsa-miR-3202 | 0.095747706 | 1.469161426 | 3.925078757 | 0.00 | 0.002106507 | 0.307076831 |
| kshv-miR-K12-3 | 0.121031222 | 2.973565135 | 3.926045196 | 0.00 | 0.002106507 | 0.310590422 |
| hsa-miR-3136-5p | 0.095602915 | 1.423493513 | 3.909747211 | 0.00 | 0.002202159 | 0.251436421 |
| hsa-miR-1261 | 0.065722347 | 1.363140322 | 3.901558749 | 0.00 | 0.002240038 | 0.221795711 |
| hsa-miR-224* | -0.115761498 | 1.585020147 | -3.885057857 | 0.00 | 0.002352594 | 0.162227411 |
| hsa-miR-592 | 0.024948458 | 1.303091888 | 3.880953856 | 0.00 | 0.002356605 | 0.147445588 |
| hsa-miR-204 | -0.218323442 | 1.75529339 | -3.872870654 | 0.00 | 0.002368165 | 0.118370671 |
| hsa-miR-221* | -0.158495521 | 1.618632165 | -3.872496671 | 0.00 | 0.002368165 | 0.11702673 |
| hsa-miR-1273e | 0.084266233 | 1.460703939 | 3.835508521 | 0.00 | 0.002689347 | -0.015341821 |
| hsa-miR-29c* | 0.126643965 | 2.012332118 | 3.826318467 | 0.00 | 0.002728362 | -0.048060362 |
| hsa-miR-514b-5p | 0.124548955 | 1.607158382 | 3.817956663 | 0.00 | 0.002728362 | -0.077771354 |
| hsa-miR-526b | 0.029174642 | 1.313942575 | 3.819867097 | 0.00 | 0.002728362 | -0.070988179 |
| hsa-miR-944 | -0.029502711 | 1.304733371 | -3.820722006 | 0.00 | 0.002728362 | -0.067951797 |
| hsa-miR-149 | -0.113564413 | 2.07862276 | -3.780447698 | 0.00 | 0.003102371 | -0.21035667 |
| hsa-miR-3189-3p | 0.035180599 | 1.305895134 | 3.773987884 | 0.00 | 0.003139075 | -0.233076328 |
| hsa-miR-148b | 0.171894646 | 2.033851912 | 3.756188106 | 0.00 | 0.003313924 | -0.295505382 |
| hsa-miR-148a | 0.205713705 | 2.988136266 | 3.749345005 | 0.00 | 0.00331855 | -0.319438075 |
| hsa-miR-4299 | 0.170974486 | 2.89471882 | 3.749625575 | 0.00 | 0.00331855 | -0.318457569 |
| hsa-miR-648 | 0.026748145 | 1.270551814 | 3.728110788 | 0.00 | 0.003548666 | -0.3934604 |
| hsa-miR-1179 | 0.031340012 | 1.330897584 | 3.701861779 | 0.00 | 0.003864804 | -0.48445923 |
| hsa-miR-1202 | 0.099214158 | 3.42559983 | 3.684507163 | 0.00 | 0.00407202 | -0.544316049 |
| hsa-miR-3616-3p | 0.020698425 | 1.309858632 | 3.681416625 | 0.00 | 0.00407202 | -0.554949739 |
| hsa-miR-939 | 0.124006715 | 2.804922747 | 3.66327045 | 0.00 | 0.004303091 | -0.617228727 |
| hur_4 | 0.061656451 | 3.68465525 | 3.65999096 | 0.00 | 0.004306608 | -0.628455518 |
| hsa-miR-222* | -0.015530495 | 1.240240552 | -3.645929933 | 0.00 | 0.004483562 | -0.676491567 |
| hsa-miR-141 | -0.126959579 | 3.301648444 | -3.642852771 | 0.00 | 0.004484823 | -0.686982386 |
| hsa-miR-29c | 0.091888432 | 3.355894506 | 3.614700152 | 0.00 | 0.00486185 | -0.782601892 |
| hsa-miR-3154 | 0.06618357 | 1.400192646 | 3.617106422 | 0.00 | 0.00486185 | -0.774454454 |
| hsa-miR-625 | 0.15190109 | 1.556932324 | 3.608126212 | 0.00 | 0.004882354 | -0.804836512 |
| hsa-miR-147b | -0.012280247 | 1.243454327 | -3.607717328 | 0.00 | 0.004882354 | -0.806218285 |
| hsa-miR-630 | 0.221252425 | 2.448308539 | 3.597586551 | 0.00 | 0.004960925 | -0.840410094 |
| hsv1-miR-H1 | 0.150223749 | 1.578398169 | 3.600217403 | 0.00 | 0.004960925 | -0.831538956 |
| ebv-miR-BART3* | 0.062708288 | 1.414232166 | 3.553412645 | 0.00 | 0.005754836 | -0.988511978 |
| hsa-miR-29a | 0.088958477 | 3.38784204 | 3.534979267 | 0.00 | 0.00608441 | -1.049837535 |
| hsa-miR-1274a_v16.0 | -0.114919463 | 2.999744957 | -3.493167689 | 0.00 | 0.006986109 | -1.18789658 |
| hsa-miR-3923 | 0.123803272 | 1.567148455 | 3.460449315 | 0.00 | 0.007760572 | -1.294917618 |
| hsa-miR-3147 | 0.093546866 | 1.454064214 | 3.434457497 | 0.00 | 0.008334407 | -1.379300206 |
| hsa-miR-4286 | -0.099266179 | 3.161057567 | -3.4360253 | 0.00 | 0.008334407 | -1.374226317 |
| hsa-miR-138-2* | 0.031134786 | 1.290633375 | 3.398954065 | 0.00 | 0.009336593 | -1.493649342 |
| hsa-miR-503 | 0.026058654 | 1.268104787 | 3.39307854 | 0.00 | 0.009437992 | -1.51247128 |
| hsa-miR-3159 | 0.016154349 | 1.24457843 | 3.381955092 | 0.00 | 0.009715037 | -1.548025281 |
| hsa-miR-193a-5p | -0.104092391 | 1.823734912 | -3.375093066 | 0.00 | 0.009795006 | -1.569906591 |
| hsa-miR-4260 | 0.021526992 | 1.282687986 | 3.374183468 | 0.00 | 0.009795006 | -1.572804103 |
| hsa-miR-1914* | -0.135498545 | 2.367429368 | -3.325902516 | 0.00 | 0.011446246 | -1.725601909 |
| hsa-miR-484 | 0.087264635 | 1.769020998 | 3.313926824 | 0.00 | 0.011813435 | -1.763197537 |
| hsa-miR-30a | 0.119378496 | 2.697777741 | 3.300567298 | 0.00 | 0.012181382 | -1.804994249 |
| hcmv-miR-US4 | 0.077732791 | 1.469238879 | 3.299228874 | 0.00 | 0.012181382 | -1.809173321 |
| hsa-miR-29b-2* | 0.024487407 | 1.279287239 | 3.296967513 | 0.00 | 0.012181382 | -1.816230706 |
| hsa-miR-378 | -0.122968544 | 2.117144264 | -3.283266507 | 0.00 | 0.012645906 | -1.858896859 |
| hsa-miR-3177-3p | 0.017407285 | 1.267656546 | 3.275616667 | 0.00 | 0.012863369 | -1.882649836 |
| hsa-miR-558 | -0.011873773 | 1.242569977 | -3.25482185 | 0.00 | 0.013673382 | -1.946967014 |
| hcmv-miR-US25-2-5p | 0.023343377 | 1.278316466 | 3.244736827 | 0.00 | 0.014021575 | -1.97802692 |
| hsa-miR-1274b_v16.0 | -0.053489564 | 3.549545813 | -3.234925036 | 0.00 | 0.014245745 | -2.008162154 |
| hsa-miR-1915 | 0.086937916 | 3.207656332 | 3.236614355 | 0.00 | 0.014245745 | -2.002979552 |
| hsv1-miR-H6-5p | 0.140180352 | 1.825224036 | 3.217973793 | 0.00 | 0.014945643 | -2.060031501 |
| hsa-miR-1290 | -0.112631837 | 1.72086801 | -3.209635631 | 0.00 | 0.015107408 | -2.085455518 |
| hsa-miR-150* | 0.131167813 | 2.305894572 | 3.208821341 | 0.00 | 0.015107408 | -2.087935199 |
| hsa-miR-760 | 0.044713284 | 1.371228209 | 3.207316089 | 0.00 | 0.015107408 | -2.092517504 |
| ebv-miR-BART10 | 0.064933281 | 1.375023051 | 3.200367508 | 0.00 | 0.015290878 | -2.113645343 |
| hsa-miR-4314 | 0.060147559 | 1.387873515 | 3.198810882 | 0.00 | 0.015290878 | -2.118372753 |
| hsa-miR-144* | 0.182673215 | 1.783349666 | 3.192270358 | 0.00 | 0.015500923 | -2.138213392 |
| hsa-miR-185 | 0.180671755 | 2.340567905 | 3.135331083 | 0.00 | 0.018526811 | -2.309388242 |
| hsa-miR-182* | -0.014982288 | 1.271448261 | -3.129718307 | 0.00 | 0.018721053 | -2.326110867 |
| hsa-miR-1285 | 0.021564437 | 1.314199756 | 3.108237172 | 0.00 | 0.019913473 | -2.389860661 |
| hsa-miR-130b | 0.125733433 | 2.282626294 | 3.082289892 | 0.00 | 0.021480777 | -2.466333493 |
| hsa-miR-610 | 0.032070333 | 1.35080018 | 3.074598548 | 0.00 | 0.021849542 | -2.488889859 |
| hsa-miR-3198 | 0.111090884 | 2.784032036 | 3.065349576 | 0.00 | 0.022335644 | -2.515946434 |
| mr_1 | 0.030078873 | 3.558522888 | 3.054380039 | 0.00 | 0.022957695 | -2.547940182 |
| hsa-miR-4298 | 0.142173297 | 2.533993918 | 3.049152458 | 0.00 | 0.023169688 | -2.563150225 |
| hsa-miR-3667-5p | 0.117162076 | 1.590021271 | 3.043199634 | 0.00 | 0.023438303 | -2.580441536 |
| hsa-miR-129-3p | -0.076030098 | 1.641621686 | -3.014516949 | 0.00 | 0.025466093 | -2.663325209 |
| hsa-miR-3132 | 0.068187396 | 1.467368088 | 3.01233542 | 0.00 | 0.025466093 | -2.669599836 |
| hsa-miR-142-5p | 0.149654392 | 2.800493378 | 2.993593047 | 0.00 | 0.026789324 | -2.723336793 |
| hsa-miR-365* | 0.027040278 | 1.294252409 | 2.991626251 | 0.00 | 0.026789324 | -2.728958112 |
| hsa-miR-30e | -0.086508164 | 2.720191699 | -2.979575014 | 0.00 | 0.027621081 | -2.763328168 |
| hsv2-miR-H3 | 0.032348691 | 1.321743005 | 2.975824445 | 0.00 | 0.027749541 | -2.773998895 |
| ebv-miR-BART12 | 0.149318589 | 2.103003788 | 2.971928499 | 0.00 | 0.027892302 | -2.78507022 |
| hsa-miR-345 | 0.062661114 | 1.379891562 | 2.957550658 | 0.00 | 0.028965702 | -2.825813685 |
| hsa-miR-95 | -0.148842767 | 1.696703149 | -2.952571625 | 0.00 | 0.029213867 | -2.839880952 |
| hsa-miR-3680* | 0.018748875 | 1.280037456 | 2.948796209 | 0.00 | 0.029216413 | -2.850533175 |
| hsa-miR-634 | -0.062513839 | 1.564994205 | -2.94697078 | 0.00 | 0.029216413 | -2.855679091 |
| hsv2-miR-H9-5p | 0.098544945 | 1.385851727 | 2.945934657 | 0.00 | 0.029216413 | -2.858598641 |
| hsa-miR-31* | -0.190758563 | 2.141725031 | -2.922572595 | 0.00 | 0.031190287 | -2.924177616 |
| hsa-miR-652 | 0.100021322 | 1.620524998 | 2.918556488 | 0.00 | 0.031367424 | -2.935402888 |
| hsa-miR-143 | 0.135794641 | 1.82904996 | 2.906073751 | 0.00 | 0.032215264 | -2.970202478 |
| hsa-miR-3180 | 0.078993228 | 1.282007054 | 2.905549634 | 0.00 | 0.032215264 | -2.971660627 |
| hsa-miR-497 | 0.149792573 | 2.85075711 | 2.899062609 | 0.00 | 0.032646567 | -2.989688203 |
| hsa-miR-127-3p | 0.100364234 | 2.33958848 | 2.871350589 | 0.00 | 0.035292498 | -3.066283446 |
| hsa-miR-92a-1* | -0.011962 | 1.240862257 | -2.850376279 | 0.00 | 0.037363753 | -3.123805778 |
| hsa-miR-181d | 0.08464422 | 1.664946798 | 2.848205439 | 0.00 | 0.037368291 | -3.12973716 |
| hsa-miR-654-5p | 0.030260122 | 1.378415153 | 2.84591661 | 0.00 | 0.037387525 | -3.135986414 |
| hsa-miR-4327 | 0.125736299 | 2.236640112 | 2.834453149 | 0.01 | 0.038455604 | -3.167215757 |
| hsa-miR-767-3p | -0.031719529 | 1.429753303 | -2.830804057 | 0.01 | 0.038635073 | -3.177132417 |
| hsa-miR-139-5p | 0.095067465 | 1.690490142 | 2.81698876 | 0.01 | 0.040017122 | -3.21456968 |
| hsa-miR-181c* | 0.042997551 | 1.352505186 | 2.806154421 | 0.01 | 0.041077033 | -3.243810756 |
| hsa-miR-181c | 0.090942087 | 1.778689619 | 2.791756021 | 0.01 | 0.042350411 | -3.282509902 |
| hsa-miR-222 | -0.090286443 | 2.349331813 | -2.793437716 | 0.01 | 0.042350411 | -3.27799943 |
| hsa-miR-4317 | -0.063632575 | 1.437616783 | -2.787960768 | 0.01 | 0.042377353 | -3.292679917 |
| hsa-miR-506 | -0.045750774 | 1.266424485 | -2.785506603 | 0.01 | 0.042377353 | -3.299249454 |
| hsa-miR-548j | -0.012400132 | 1.255937295 | -2.785399933 | 0.01 | 0.042377353 | -3.299534875 |
| hsa-miR-219-2-3p | 0.012915099 | 1.263039179 | 2.775125425 | 0.01 | 0.043425543 | -3.326979572 |
| hsa-miR-200b* | -0.041372949 | 1.368857945 | -2.772340331 | 0.01 | 0.043524074 | -3.334402799 |
| hsa-miR-99a | -0.126913462 | 2.957789834 | -2.749544917 | 0.01 | 0.046272978 | -3.394901005 |
| ebv-miR-BART14* | -0.012900522 | 1.230900887 | -2.742154175 | 0.01 | 0.046935448 | -3.414416448 |
| jcv-miR-J1-5p | 0.016129557 | 1.264694511 | 2.740690817 | 0.01 | 0.046935448 | -3.418274708 |
| hsa-miR-520b | -0.056331407 | 1.371422028 | -2.728724333 | 0.01 | 0.048325228 | -3.449753656 |

DEmiRNA- Differentially expressed miRNA, logFC- Log Fold Change, AveExpr- Average Expression, adj.P.Val- Adjusted P value

**Table S1b: Significant DEmiRNA in H.pylori-infected gastric tissue (GSE32174)**

| **miRNA** | **logFC** | **AveExpr** | **t** | **P Value** | **adj.P.Val** | **B** |
| --- | --- | --- | --- | --- | --- | --- |
| hsa-miR-155 | 1.170682717 | 13.21967681 | 11.02882854 | 1.01E-14 | 4.29E-12 | 23.34938374 |
| hsa-miR-519e | 1.206927197 | 7.693870618 | 9.333986149 | 2.44E-12 | 5.18E-10 | 17.9229067 |
| hsa-miR-650 | 4.011519666 | 9.459744964 | 8.974080221 | 8.16E-12 | 1.16E-09 | 16.72637633 |
| hsa-miR-146b-5p | 0.600843623 | 13.65718416 | 8.603440585 | 2.87E-11 | 3.05E-09 | 15.48008079 |
| hsa-miR-146a | 0.702225251 | 14.44323395 | 8.532563716 | 3.66E-11 | 3.11E-09 | 15.24024225 |
| hsa-miR-9* | 2.064600403 | 10.95742594 | 8.202634869 | 1.14E-10 | 8.06E-09 | 14.11797136 |
| hsa-miR-223 | 0.56152424 | 14.47269781 | 7.920204628 | 3.03E-10 | 1.84E-08 | 13.15038159 |
| hsa-miR-150 | 0.358675695 | 14.5565064 | 6.972330984 | 8.35E-09 | 4.44E-07 | 9.870707888 |
| HS_163 | 2.805203408 | 9.041346891 | 6.614749071 | 2.94E-08 | 1.39E-06 | 8.627792289 |
| hsa-miR-190 | -0.657694298 | 9.442575283 | -5.675328542 | 7.96E-07 | 3.38E-05 | 5.383273426 |
| hsa-miR-9 | 1.450102985 | 10.13888198 | 5.556228927 | 1.20E-06 | 4.65E-05 | 4.977031689 |
| hsa-miR-660 | -0.480142157 | 12.08113518 | -5.530172337 | 1.32E-06 | 4.67E-05 | 4.888384546 |
| hsa-miR-582-5p | -0.390560262 | 11.4995218 | -5.413715337 | 1.97E-06 | 6.45E-05 | 4.493287845 |
| hsa-miR-618 | 1.155375728 | 7.387401974 | 5.387809934 | 2.16E-06 | 6.55E-05 | 4.405658289 |
| hsa-miR-766 | 0.861626152 | 10.77586261 | 5.351826421 | 2.44E-06 | 6.93E-05 | 4.284102322 |
| hsa-miR-142-3p | 0.240897861 | 14.26279205 | 5.320633788 | 2.72E-06 | 7.23E-05 | 4.178889612 |
| hsa-miR-196b | 0.54648464 | 9.333412256 | 4.888461842 | 1.19E-05 | 0.000297671 | 2.738705016 |
| hsa-miR-193b | 0.389792642 | 13.45253724 | 4.813381364 | 1.53E-05 | 0.000339014 | 2.492335623 |
| hsa-miR-652 | -0.39259766 | 11.79105474 | -4.806620492 | 1.57E-05 | 0.000339014 | 2.470213318 |
| hsa-miR-200a* | -0.40489765 | 12.09988888 | -4.801682542 | 1.60E-05 | 0.000339014 | 2.454062504 |
| hsa-miR-502-3p, | -0.285361316 | 12.35480895 | -4.626064802 | 2.87E-05 | 0.000554593 | 1.883492701 |
| hsa-miR-532-5p | -0.288075506 | 12.57141846 | -4.628845636 | 2.84E-05 | 0.000554593 | 1.892467034 |
| hsa-miR-455-5p | -0.425811459 | 11.96651249 | -4.551201002 | 3.68E-05 | 0.00068008 | 1.642662033 |
| hsa-miR-204 | -0.760201696 | 11.53522364 | -4.245479211 | 0.000100083 | 0.001772305 | 0.675875463 |
| hsa-miR-34a | 0.436488942 | 13.75954001 | 4.202419707 | 0.000114998 | 0.001954966 | 0.542049099 |
| HS_282 | 0.76529463 | 7.684305379 | 4.121957191 | 0.000148869 | 0.002433438 | 0.293654179 |
| hsa-miR-137 | -0.728552543 | 7.751115477 | -4.07705049 | 0.000171797 | 0.002704206 | 0.1560029 |
| hsa-miR-508-3p | 0.458411508 | 7.296977451 | 3.993747948 | 0.000223718 | 0.003395727 | -0.097413824 |
| hsa-miR-122 | -0.734700767 | 6.954561131 | -3.98139727 | 0.000232608 | 0.003408912 | -0.134767575 |
| hsa-miR-95 | -0.318081634 | 12.28322805 | -3.947515095 | 0.000258786 | 0.003666137 | -0.236945544 |
| hsa-miR-594:9.1 | 0.238631401 | 13.50084369 | 3.934953747 | 0.000269197 | 0.003690609 | -0.274715169 |
| hsa-miR-768-3p:11.0 | 0.187825606 | 14.1698848 | 3.87835785 | 0.000321343 | 0.004267834 | -0.444127772 |
| HS_279_a | 0.306044863 | 7.794664686 | 3.859587387 | 0.000340698 | 0.004387772 | -0.500035777 |
| hsa-miR-801:9.1 | 0.93761032 | 9.922906351 | 3.831881437 | 0.000371335 | 0.004572351 | -0.582299945 |
| hsa-miR-30e* | -0.141774459 | 13.8499489 | -3.827388828 | 0.000376547 | 0.004572351 | -0.59561009 |
| hsa-miR-362-5p | -0.507549638 | 11.44445485 | -3.684229442 | 0.000584892 | 0.006904979 | -1.015347393 |
| hsa-miR-342-3p | 0.197246297 | 13.32538876 | 3.648394302 | 0.000652292 | 0.007333426 | -1.119040833 |
| hsa-miR-501-5p | -0.45135382 | 10.58758405 | -3.646680801 | 0.000655695 | 0.007333426 | -1.123984894 |
| hsa-miR-30a | -0.214979931 | 13.85630947 | -3.50874403 | 0.000993086 | 0.010822088 | -1.517624439 |
| hsa-miR-151:9.1 | -0.1810052 | 13.33738661 | -3.486269157 | 0.001061823 | 0.011281874 | -1.580927068 |
| hsa-miR-181b | 0.192574752 | 12.99671783 | 3.4717385 | 0.001108656 | 0.011492163 | -1.621726217 |
| hsa-miR-365 | 0.26534018 | 12.09764324 | 3.377993025 | 0.001461561 | 0.014789607 | -1.882485373 |
| hsa-miR-196a | 1.052522822 | 8.488883863 | 3.344760048 | 0.001610565 | 0.015210896 | -1.973881915 |
| hsa-miR-143 | 0.203159125 | 14.56643776 | 3.355921708 | 0.001558981 | 0.015210896 | -1.943247279 |
| hsa-miR-484 | 0.135604494 | 13.37739722 | 3.346127882 | 0.001604158 | 0.015210896 | -1.970131097 |
| hsa-miR-29a | 0.122678809 | 14.00514321 | 3.318356893 | 0.001739114 | 0.015726027 | -2.046097563 |
| HS_176 | -0.475719609 | 8.031922133 | -3.325366848 | 0.001704066 | 0.015726027 | -2.026959119 |
| hsa-miR-642 | -0.297204496 | 13.21982505 | -3.303726872 | 0.001814472 | 0.016065639 | -2.085959163 |
| HS_169 | 0.85469086 | 8.91424172 | 3.267342013 | 0.002015532 | 0.017481656 | -2.184616262 |
| hsa-miR-431 | 0.630347158 | 8.293929748 | 3.235775047 | 0.002206881 | 0.018758491 | -2.269650271 |
| HS_243.1 | 0.671019131 | 10.59702854 | 3.186642098 | 0.002539211 | 0.021160094 | -2.400952424 |
| hsa-miR-30d | -0.107366151 | 14.44853013 | -3.1638524 | 0.002708901 | 0.022140055 | -2.46141499 |
| hsa-miR-28-5p | -0.119872201 | 13.83622023 | -3.13974931 | 0.002899966 | 0.023254446 | -2.525054619 |
| hsa-miR-502-5p | -0.517722733 | 7.318292863 | -3.109955021 | 0.00315369 | 0.024820706 | -2.603279576 |
| HS_182.1 | 0.539568509 | 7.023839851 | 3.05935537 | 0.003632982 | 0.027199458 | -2.734996242 |
| hsa-miR-212 | 0.361854609 | 10.59655402 | 3.064196665 | 0.003584325 | 0.027199458 | -2.722456086 |
| hsa-miR-152 | 0.243760507 | 13.5890127 | 3.047955706 | 0.003750013 | 0.027199458 | -2.764471733 |
| HS_114 | -0.325439088 | 7.576386831 | -3.050397955 | 0.003724647 | 0.027199458 | -2.758163154 |
| hsa-miR-30a* | -0.354382864 | 11.72305459 | -3.039422015 | 0.003839923 | 0.027199458 | -2.786488481 |
| hsa-miR-153 | -0.463949984 | 11.49414713 | -3.044139441 | 0.003789974 | 0.027199458 | -2.774322746 |
| hsa-miR-565:9.1 | 0.290180719 | 13.67129573 | 3.017478149 | 0.004080485 | 0.028429611 | -2.842912077 |
| hsa-miR-324-5p | -0.129408038 | 12.61775804 | -3.003027557 | 0.004246529 | 0.029109273 | -2.87991731 |
| HS_188 | 0.725782926 | 10.5293755 | 2.988651918 | 0.004417966 | 0.029803742 | -2.916610759 |
| hsa-miR-132 | 0.16060594 | 13.68965954 | 2.92045041 | 0.005323053 | 0.0353484 | -3.089045067 |
| hsa-miR-665 | 0.366673212 | 8.15609142 | 2.910294755 | 0.005471752 | 0.035776837 | -3.114486129 |
| hsa-miR-487a | 0.594920393 | 8.060657012 | 2.882851747 | 0.005893135 | 0.03794822 | -3.18292463 |
| HS_5.1 | 0.616611667 | 8.109877905 | 2.863436965 | 0.006209259 | 0.038652856 | -3.231067419 |
| hsa-miR-625 | 0.157694645 | 13.69811408 | 2.859491055 | 0.006275405 | 0.038652856 | -3.240824093 |
| hsa-miR-29b | 0.117628398 | 14.40380947 | 2.863736257 | 0.006204268 | 0.038652856 | -3.230327003 |
| hsa-miR-651 | -0.284692208 | 9.51316203 | -2.832347685 | 0.006748439 | 0.040972665 | -3.307681117 |
| hsa-miR-138 | 0.831628961 | 9.242975313 | 2.826623774 | 0.006852321 | 0.041015794 | -3.321722003 |
| hsa-miR-224 | 0.581181041 | 10.87313285 | 2.816213396 | 0.007045066 | 0.041015794 | -3.347207112 |
| hsa-miR-96 | -0.494000276 | 11.00293669 | -2.819355576 | 0.006986367 | 0.041015794 | -3.339521957 |
| hsa-miR-671:9.1 | 0.568762955 | 7.862186068 | 2.79767088 | 0.00740086 | 0.042190586 | -3.392433882 |
| hsa-let-7c | -0.21986668 | 13.01012961 | -2.795408166 | 0.007445397 | 0.042190586 | -3.397938206 |
| hsa-miR-345:9.1 | -0.301723514 | 10.51702801 | -2.775703358 | 0.007843861 | 0.043863699 | -3.4457374 |
| hsa-miR-566 | 0.70054793 | 7.570540767 | 2.755224795 | 0.00827878 | 0.045108735 | -3.495155404 |
| hsa-miR-182 | -0.143062238 | 12.6742003 | -2.757137902 | 0.008237226 | 0.045108735 | -3.490549965 |
| hsa-miR-554 | 0.27375096 | 8.306235282 | 2.7269616 | 0.00891563 | 0.047364287 | -3.562923438 |
| hsa-miR-183 | -0.248465157 | 12.20547505 | -2.731455634 | 0.008811437 | 0.047364287 | -3.552181815 |
| hsa-miR-338-3p | -0.232512997 | 13.51347154 | -2.721824016 | 0.009036134 | 0.047411814 | -3.575187491 |
| hsa-miR-181a* | 0.245686468 | 11.01001424 | 2.708815768 | 0.009347984 | 0.048449917 | -3.606164327 |

DEmiRNA- Differentially expressed miRNA, logFC- Log Fold Change, AveExpr- Average Expression, adj.P.Val- Adjusted P value

**Table S2a. shared-DEmiRNA-gene network-interactions**

| **Node** | **Degree** | **Betweenness** |
| --- | --- | --- |
| hsa-mir-484 | 69 | 1962.136 |
| hsa-mir-155-5p | 66 | 1636.317 |
| hsa-mir-29a-3p | 50 | 1017.875 |
| hsa-mir-30a-5p | 50 | 856.5797 |
| hsa-mir-143-3p | 37 | 588.0823 |
| hsa-mir-204-5p | 32 | 379.1202 |
| hsa-mir-625-5p | 29 | 435.7028 |
| hsa-mir-30a-3p | 28 | 280.4113 |
| hsa-mir-29a-5p | 21 | 167.7939 |
| hsa-mir-650 | 19 | 324.8615 |
| hsa-mir-652-3p | 19 | 123.6387 |
| hsa-mir-223-3p | 17 | 102.9755 |
| hsa-mir-143-5p | 14 | 114.1291 |
| hsa-mir-223-5p | 14 | 98.76165 |
| hsa-mir-625-3p | 13 | 125.2958 |
| hsa-mir-652-5p | 13 | 93.50127 |
| hsa-mir-95-5p | 13 | 47.12287 |
| hsa-mir-155-3p | 12 | 97.93523 |
| hsa-mir-204-3p | 12 | 68.5316 |
| hsa-mir-95-3p | 12 | 44.82033 |
| PTEN | 11 | 137.5331 |
| CCND1 | 11 | 123.9173 |
| hsa-mir-204 | 11 | 83.92653 |
| MDM2 | 10 | 71.53003 |
| TNRC6A | 9 | 152.6739 |
| SCD | 9 | 80.50235 |
| SLC7A5 | 9 | 79.59176 |
| ZNF460 | 9 | 68.25678 |
| CBX5 | 9 | 66.1374 |
| CLTC | 9 | 59.27462 |
| CDK6 | 8 | 66.14772 |
| CANX | 8 | 63.33271 |
| SMAD2 | 8 | 62.21069 |
| SOX4 | 8 | 58.47482 |
| CCNT2 | 8 | 48.09324 |
| AGO2 | 8 | 46.94585 |
| STMN1 | 7 | 66.09744 |
| FBXW7 | 7 | 62.18554 |
| GLUL | 7 | 56.15493 |
| PEG10 | 7 | 54.78503 |
| ATF7IP | 7 | 52.88426 |
| OAZ1 | 7 | 52.32506 |
| IGF1R | 7 | 40.56438 |
| ELK4 | 7 | 34.01685 |
| DDX6 | 7 | 32.92598 |
| ABL1 | 7 | 28.90191 |
| hsa-mir-29a | 7 | 15.33121 |
| DNMT3A | 6 | 91.72211 |
| LDLR | 6 | 42.70336 |
| BTG2 | 6 | 42.37793 |
| NFIA | 6 | 42.16979 |
| RHOB | 6 | 39.11959 |
| MYH9 | 6 | 37.32845 |
| ATN1 | 6 | 37.28873 |
| PARP1 | 6 | 36.04237 |
| PARP1 | 6 | 36.04237 |
| AP1G1 | 6 | 35.78766 |
| CREBRF | 6 | 35.42628 |
| SRCAP | 6 | 34.8772 |
| PURA | 6 | 32.52788 |
| HYOU1 | 6 | 32.44648 |
| FLNA | 6 | 32.39668 |
| COL3A1 | 6 | 29.2467 |
| ZNF711 | 6 | 29.17192 |
| POLR2A | 6 | 28.18559 |
| ADAR | 6 | 26.72495 |
| BRD2 | 6 | 26.10975 |
| ITM2B | 6 | 25.96112 |
| SKI | 6 | 24.71064 |
| SPAG9 | 6 | 24.37453 |
| EEF2 | 6 | 23.96346 |
| ZEB1 | 6 | 23.6821 |
| SUCO | 6 | 22.97188 |
| CAPRIN1 | 6 | 22.9374 |
| hsa-mir-155 | 6 | 11.39172 |
| KRAS | 5 | 74.50811 |
| ARPC1B | 5 | 32.88645 |
| SPARC | 5 | 30.97363 |
| ARHGDIA | 5 | 30.41686 |
| CDC42 | 5 | 27.49715 |
| ATP2B1 | 5 | 25.91985 |
| JARID2 | 5 | 25.40245 |
| SERBP1 | 5 | 24.84877 |
| RCC2 | 5 | 22.8464 |
| TFAM | 5 | 20.57544 |
| TSC22D3 | 5 | 20.37171 |
| HDGF | 5 | 19.96142 |
| TP53 | 5 | 19.68239 |
| ZNF226 | 5 | 19.041 |
| YY1 | 5 | 18.21037 |
| BAG2 | 5 | 16.10162 |
| RUNX2 | 5 | 12.72529 |
| hsa-mir-223 | 5 | 11.38911 |
| BACH1 | 4 | 24.59231 |
| MEIS1 | 4 | 21.89032 |
| ITGB4 | 4 | 19.98296 |
| FOXO3 | 4 | 17.01618 |
| ECE1 | 4 | 16.94056 |
| FBN2 | 4 | 16.38662 |
| HMGA2 | 4 | 15.73834 |
| CMPK1 | 4 | 15.61125 |
| MTR | 4 | 15.27875 |
| ANXA11 | 4 | 15.0904 |
| DNMT3B | 4 | 15.04026 |
| HNRNPA0 | 4 | 14.75604 |
| MMP9 | 4 | 14.70741 |
| OCRL | 4 | 14.1332 |
| IFNAR2 | 4 | 14.09237 |
| ATP1A1 | 4 | 13.38075 |
| ATP2A2 | 4 | 13.31319 |
| FJX1 | 4 | 12.11957 |
| MAT2A | 4 | 11.81762 |
| ACTB | 4 | 11.72249 |
| ACLY | 4 | 9.325001 |
| DST | 4 | 8.175454 |
| NTRK3 | 3 | 131.2241 |
| BDNF | 3 | 25.82475 |
| CDC25B | 3 | 18.94107 |
| ERF | 3 | 11.53544 |
| IRS1 | 3 | 11.13803 |
| ABCF1 | 3 | 10.95304 |
| ANKRD36 | 3 | 9.264592 |
| RBM23 | 3 | 7.840309 |
| FOXO1 | 3 | 7.289545 |
| SNRPD3 | 3 | 6.653688 |
| EDA2R | 3 | 6.136165 |
| EFNB1 | 2 | 5.554032 |
| H2AFX | 2 | 3.607107 |
| ANXA6 | 2 | 3.471201 |
| MRVI1 | 2 | 2.768769 |

**Table S2b: KEGG Pathway Enrichment Analysis of shared-DEmiRNA-gene network**

| **KEGG Pathway** | **Total** | **Expected** | **Hits** | **P value** |
| --- | --- | --- | --- | --- |
| Glioma | 65 | 0.694 | 7 | 4.48E-06 |
| Neurotrophin signaling pathway | 123 | 1.31 | 9 | 4.51E-06 |
| Prostate cancer | 87 | 0.929 | 7 | 3.14E-05 |
| Pathways in cancer | 310 | 3.31 | 12 | 7.11E-05 |
| Melanoma | 68 | 0.726 | 6 | 7.23E-05 |
| Chronic myeloid leukemia | 73 | 0.78 | 6 | 0.000108 |
| Bladder cancer | 29 | 0.31 | 4 | 0.000225 |
| Cell cycle | 124 | 1.32 | 7 | 3.00E-04 |
| Cysteine and methionine metabolism | 34 | 0.363 | 4 | 0.000422 |
| p53 signaling pathway | 68 | 0.726 | 5 | 0.000713 |
| Pancreatic cancer | 69 | 0.737 | 5 | 0.000762 |
| Salmonella infection | 72 | 0.769 | 5 | 0.000927 |
| Focal adhesion | 200 | 2.14 | 8 | 0.0011 |
| Non-small cell lung cancer | 52 | 0.556 | 4 | 0.00214 |
| Aldosterone-regulated sodium reabsorption | 34 | 0.363 | 3 | 0.00544 |
| Adherens junction | 70 | 0.748 | 4 | 0.0063 |
| MAPK signaling pathway | 265 | 2.83 | 8 | 0.0064 |
| Tight junction | 118 | 1.26 | 5 | 0.00802 |
| Endometrial cancer | 44 | 0.47 | 3 | 0.0112 |
| Regulation of actin cytoskeleton | 182 | 1.94 | 6 | 0.0122 |
| Shigellosis | 47 | 0.502 | 3 | 0.0134 |
| Colorectal cancer | 49 | 0.524 | 3 | 0.015 |
| HTLV-I infection | 199 | 2.13 | 6 | 0.0183 |
| Bacterial invasion of epithelial cells | 56 | 0.598 | 3 | 0.0214 |
| Hypertrophic cardiomyopathy (HCM) | 25 | 0.267 | 2 | 0.0287 |
| Viral myocarditis | 26 | 0.278 | 2 | 0.0309 |
| Thyroid cancer | 28 | 0.299 | 2 | 0.0354 |
| Axon guidance | 118 | 1.26 | 4 | 0.0363 |
| Dilated cardiomyopathy | 78 | 0.833 | 3 | 0.0499 |
| Progesterone-mediated oocyte maturation | 80 | 0.855 | 3 | 0.0531 |
| Small cell lung cancer | 80 | 0.855 | 3 | 0.0531 |
| Pathogenic Escherichia coli infection | 35 | 0.374 | 2 | 0.0532 |
| Endocrine and other factor-regulated calcium reabsorption | 36 | 0.385 | 2 | 0.056 |
| Mineral absorption | 8 | 0.0855 | 1 | 0.0824 |
| Hepatitis C | 100 | 1.07 | 3 | 0.0903 |
| Endocytosis | 101 | 1.08 | 3 | 0.0924 |
| Measles | 102 | 1.09 | 3 | 0.0945 |
| Leukocyte transendothelial migration | 108 | 1.15 | 3 | 0.108 |
| Selenocompound metabolism | 12 | 0.128 | 1 | 0.121 |
| Dorso-ventral axis formation | 12 | 0.128 | 1 | 0.121 |
| Acute myeloid leukemia | 57 | 0.609 | 2 | 0.123 |
| Inositol phosphate metabolism | 58 | 0.62 | 2 | 0.127 |
| RNA degradation | 60 | 0.641 | 2 | 0.134 |
| Long-term depression | 70 | 0.748 | 2 | 0.171 |
| Synaptic vesicle cycle | 18 | 0.192 | 1 | 0.176 |
| Insulin signaling pathway | 137 | 1.46 | 3 | 0.179 |
| One carbon pool by folate | 19 | 0.203 | 1 | 0.185 |
| Vibrio cholerae infection | 19 | 0.203 | 1 | 0.185 |
| Phosphatidylinositol signaling system | 75 | 0.801 | 2 | 0.191 |
| VEGF signaling pathway | 76 | 0.812 | 2 | 0.195 |
| Vasopressin-regulated water reabsorption | 22 | 0.235 | 1 | 0.211 |
| ECM-receptor interaction | 84 | 0.897 | 2 | 0.226 |
| ErbB signaling pathway | 87 | 0.929 | 2 | 0.238 |
| Epstein-Barr virus infection | 91 | 0.972 | 2 | 0.254 |
| Huntington's disease | 28 | 0.299 | 1 | 0.26 |
| Alcoholism | 166 | 1.77 | 3 | 0.261 |
| GnRH signaling pathway | 94 | 1 | 2 | 0.266 |
| Citrate cycle (TCA cycle) | 30 | 0.321 | 1 | 0.276 |
| Fc gamma R-mediated phagocytosis | 97 | 1.04 | 2 | 0.278 |
| T cell receptor signaling pathway | 98 | 1.05 | 2 | 0.282 |
| Jak-STAT signaling pathway | 99 | 1.06 | 2 | 0.286 |
| Alanine, aspartate and glutamate metabolism | 32 | 0.342 | 1 | 0.292 |
| Pyrimidine metabolism | 101 | 1.08 | 2 | 0.294 |
| Chemokine signaling pathway | 189 | 2.02 | 3 | 0.329 |
| Cocaine addiction | 43 | 0.459 | 1 | 0.371 |
| Type II diabetes mellitus | 48 | 0.513 | 1 | 0.404 |
| Natural killer cell mediated cytotoxicity | 138 | 1.47 | 2 | 0.437 |
| Arginine and proline metabolism | 56 | 0.598 | 1 | 0.454 |
| Gastric acid secretion | 56 | 0.598 | 1 | 0.454 |
| Wnt signaling pathway | 144 | 1.54 | 2 | 0.459 |
| Renal cell carcinoma | 60 | 0.641 | 1 | 0.477 |
| Adipocytokine signaling pathway | 63 | 0.673 | 1 | 0.494 |
| PPAR signaling pathway | 64 | 0.684 | 1 | 0.499 |
| Long-term potentiation | 70 | 0.748 | 1 | 0.531 |
| B cell receptor signaling pathway | 75 | 0.801 | 1 | 0.556 |
| Fc epsilon RI signaling pathway | 75 | 0.801 | 1 | 0.556 |
| Calcium signaling pathway | 177 | 1.89 | 2 | 0.57 |
| Apoptosis | 83 | 0.887 | 1 | 0.593 |
| TGF-beta signaling pathway | 84 | 0.897 | 1 | 0.598 |
| Serotonergic synapse | 88 | 0.94 | 1 | 0.615 |
| Gap junction | 89 | 0.951 | 1 | 0.619 |
| Chagas disease (American trypanosomiasis) | 89 | 0.951 | 1 | 0.619 |
| Cholinergic synapse | 95 | 1.01 | 1 | 0.643 |
| Toll-like receptor signaling pathway | 97 | 1.04 | 1 | 0.651 |
| Melanogenesis | 101 | 1.08 | 1 | 0.666 |
| Herpes simplex infection | 103 | 1.1 | 1 | 0.673 |
| Influenza A | 107 | 1.14 | 1 | 0.687 |
| Oocyte meiosis | 108 | 1.15 | 1 | 0.691 |
| Vascular smooth muscle contraction | 109 | 1.16 | 1 | 0.694 |
| Osteoclast differentiation | 119 | 1.27 | 1 | 0.726 |
| Protein processing in endoplasmic reticulum | 129 | 1.38 | 1 | 0.755 |
| Cytokine-cytokine receptor interaction | 253 | 2.7 | 2 | 0.762 |
| Purine metabolism | 163 | 1.74 | 1 | 0.832 |

**Table S2c: Reactome Pathway Enrichment Analsysis of shared-DEmiRNA-gene network**

| **Reactome Pathway** | **Total** | **Expected** | **Hits** | **P value** |
| --- | --- | --- | --- | --- |
| Signaling by SCF-KIT | 133 | 1.48 | 9 | 1.43E-05 |
| Oncogene Induced Senescence | 30 | 0.333 | 5 | 1.70E-05 |
| Pre-NOTCH Transcription and Translation | 19 | 0.211 | 4 | 4.81E-05 |
| PI3K events in ERBB4 signaling | 94 | 1.04 | 7 | 7.45E-05 |
| PIP3 activates AKT signaling | 94 | 1.04 | 7 | 7.45E-05 |
| PI3K events in ERBB2 signaling | 94 | 1.04 | 7 | 7.45E-05 |
| PI-3K cascade:FGFR1 | 94 | 1.04 | 7 | 7.45E-05 |
| PI-3K cascade:FGFR2 | 94 | 1.04 | 7 | 7.45E-05 |
| PI-3K cascade:FGFR3 | 94 | 1.04 | 7 | 7.45E-05 |
| PI-3K cascade:FGFR4 | 94 | 1.04 | 7 | 7.45E-05 |
| Signaling by EGFR | 168 | 1.87 | 9 | 9.08E-05 |
| PI3K/AKT activation | 97 | 1.08 | 7 | 9.11E-05 |
| GAB1 signalosome | 98 | 1.09 | 7 | 9.72E-05 |
| Pre-NOTCH Expression and Processing | 24 | 0.267 | 4 | 0.000126 |
| Role of LAT2/NTAL/LAB on calcium mobilization | 103 | 1.14 | 7 | 0.000133 |
| Signaling by PDGF | 177 | 1.97 | 9 | 0.000136 |
| Downstream signaling of activated FGFR1 | 139 | 1.54 | 8 | 0.00014 |
| Downstream signaling of activated FGFR2 | 139 | 1.54 | 8 | 0.00014 |
| Downstream signaling of activated FGFR3 | 139 | 1.54 | 8 | 0.00014 |
| Downstream signaling of activated FGFR4 | 139 | 1.54 | 8 | 0.00014 |
| Signaling by ERBB4 | 143 | 1.59 | 8 | 0.00017 |
| Downstream signal transduction | 151 | 1.68 | 8 | 0.000247 |
| Signaling by FGFR | 151 | 1.68 | 8 | 0.000247 |
| Signaling by FGFR1 | 151 | 1.68 | 8 | 0.000247 |
| Signaling by FGFR2 | 151 | 1.68 | 8 | 0.000247 |
| Signaling by FGFR3 | 151 | 1.68 | 8 | 0.000247 |
| Signaling by FGFR4 | 151 | 1.68 | 8 | 0.000247 |
| Signaling by ERBB2 | 152 | 1.69 | 8 | 0.000259 |
| Developmental Biology | 438 | 4.87 | 14 | 0.000275 |
| DAP12 signaling | 154 | 1.71 | 8 | 0.000283 |
| Downstream signaling events of B Cell Receptor (BCR) | 164 | 1.82 | 8 | 0.000433 |
| Fc epsilon receptor (FCERI) signaling | 169 | 1.88 | 8 | 0.00053 |
| DAP12 interactions | 171 | 1.9 | 8 | 0.000573 |
| NGF signalling via TRKA from the plasma membrane | 189 | 2.1 | 8 | 0.00111 |
| Signaling by the B Cell Receptor (BCR) | 190 | 2.11 | 8 | 0.00114 |
| Hemostasis | 450 | 5 | 13 | 0.00121 |
| Transcriptional activity of SMAD2/SMAD3:SMAD4 heterotrimer | 43 | 0.478 | 4 | 0.00125 |
| Downregulation of SMAD2/3:SMAD4 transcriptional activity | 22 | 0.244 | 3 | 0.00174 |
| Constitutive Signaling by AKT1 E17K in Cancer | 22 | 0.244 | 3 | 0.00174 |
| Gene Expression | 851 | 9.46 | 19 | 0.00191 |
| RHO GTPases activate PAKs | 23 | 0.256 | 3 | 0.00199 |
| CDO in myogenesis | 24 | 0.267 | 3 | 0.00226 |
| Myogenesis | 24 | 0.267 | 3 | 0.00226 |
| EPH-Ephrin signaling | 84 | 0.933 | 5 | 0.00231 |
| AKT phosphorylates targets in the nucleus | 7 | 0.0778 | 2 | 0.00247 |
| Post-transcriptional silencing by small RNAs | 7 | 0.0778 | 2 | 0.00247 |
| Adaptive Immune System | 430 | 4.78 | 12 | 0.00255 |
| Signaling by NOTCH | 86 | 0.956 | 5 | 0.00256 |
| Signalling by NGF | 273 | 3.03 | 9 | 0.00307 |
| RHO GTPases Activate WASPs and WAVEs | 30 | 0.333 | 3 | 0.00431 |
| Axon guidance | 292 | 3.24 | 9 | 0.00479 |
| Generic Transcription Pathway | 189 | 2.1 | 7 | 0.00481 |
| Cellular Senescence | 143 | 1.59 | 6 | 0.00494 |
| Methylation | 10 | 0.111 | 2 | 0.00518 |
| Reduction of cytosolic Ca++ levels | 10 | 0.111 | 2 | 0.00518 |
| Signaling by TGF-beta Receptor Complex | 72 | 0.8 | 4 | 0.00819 |
| Ion transport by P-type ATPases | 39 | 0.433 | 3 | 0.00904 |
| Assembly of collagen fibrils and other multimeric structures | 40 | 0.445 | 3 | 0.0097 |
| Binding and Uptake of Ligands by Scavenger Receptors | 40 | 0.445 | 3 | 0.0097 |
| EPHB-mediated forward signaling | 40 | 0.445 | 3 | 0.0097 |
| SOS-mediated signalling | 14 | 0.156 | 2 | 0.0102 |
| PRC2 methylates histones and DNA | 42 | 0.467 | 3 | 0.0111 |
| Factors involved in megakaryocyte development and platelet production | 122 | 1.36 | 5 | 0.0112 |
| RHO GTPases activate CIT | 15 | 0.167 | 2 | 0.0117 |
| RHO GTPases activate IQGAPs | 15 | 0.167 | 2 | 0.0117 |
| PI3K/AKT Signaling in Cancer | 81 | 0.9 | 4 | 0.0123 |
| RHO GTPases Activate ROCKs | 16 | 0.178 | 2 | 0.0132 |
| EPH-ephrin mediated repulsion of cells | 46 | 0.511 | 3 | 0.0142 |
| Signaling by Rho GTPases | 349 | 3.88 | 9 | 0.0147 |
| RHO GTPase Effectors | 234 | 2.6 | 7 | 0.0148 |
| Diseases of signal transduction | 235 | 2.61 | 7 | 0.0151 |
| Vesicle-mediated transport | 184 | 2.04 | 6 | 0.0161 |
| Oxidative Stress Induced Senescence | 88 | 0.978 | 4 | 0.0162 |
| SHC-related events triggered by IGF1R | 18 | 0.2 | 2 | 0.0166 |
| Signaling by NODAL | 19 | 0.211 | 2 | 0.0184 |
| Platelet calcium homeostasis | 19 | 0.211 | 2 | 0.0184 |
| Cell-extracellular matrix interactions | 19 | 0.211 | 2 | 0.0184 |
| Regulation of actin dynamics for phagocytic cup formation | 52 | 0.578 | 3 | 0.0197 |
| Platelet homeostasis | 54 | 0.6 | 3 | 0.0218 |
| Signaling by Leptin | 21 | 0.233 | 2 | 0.0223 |
| Cellular responses to stress | 256 | 2.84 | 7 | 0.023 |
| Sema4D induced cell migration and growth-cone collapse | 22 | 0.244 | 2 | 0.0244 |
| RHO GTPases Activate Formins | 102 | 1.13 | 4 | 0.0264 |
| MicroRNA (miRNA) biogenesis | 23 | 0.256 | 2 | 0.0265 |
| Signaling by Robo receptor | 23 | 0.256 | 2 | 0.0265 |
| Sema4D in semaphorin signaling | 25 | 0.278 | 2 | 0.031 |
| Lysosome Vesicle Biogenesis | 25 | 0.278 | 2 | 0.031 |
| Disease | 669 | 7.43 | 13 | 0.0315 |
| Synthesis of IP3 and IP4 in the cytosol | 27 | 0.3 | 2 | 0.0358 |
| Golgi Associated Vesicle Biogenesis | 27 | 0.3 | 2 | 0.0358 |
| SMAD2/SMAD3:SMAD4 heterotrimer regulates transcription | 28 | 0.311 | 2 | 0.0382 |
| Collagen formation | 70 | 0.778 | 3 | 0.0425 |
| Transcriptional regulation of pluripotent stem cells | 30 | 0.333 | 2 | 0.0434 |
| Cyclin D associated events in G1 | 30 | 0.333 | 2 | 0.0434 |
| G1 Phase | 30 | 0.333 | 2 | 0.0434 |
| Fcgamma receptor (FCGR) dependent phagocytosis | 72 | 0.8 | 3 | 0.0455 |
| Elongation arrest and recovery | 31 | 0.344 | 2 | 0.046 |
| HIV elongation arrest and recovery | 31 | 0.344 | 2 | 0.046 |
| Pausing and recovery of HIV elongation | 31 | 0.344 | 2 | 0.046 |
| Transcriptional regulation by small RNAs | 73 | 0.811 | 3 | 0.0471 |

**Table S2d. GO –Biologial Pathways enrichment analysis of shared-DEmiRNA-gene network**

| **GO Biological Pathway** | **Total** | **Expected** | **Hits** | **P value** |
| --- | --- | --- | --- | --- |
| negative regulation of metabolic process | 1820 | 13.1 | 37 | 1.46E-09 |
| negative regulation of cellular metabolic process | 1660 | 12 | 35 | 1.96E-09 |
| negative regulation of transcription from RNA polymerase II promoter | 552 | 3.98 | 20 | 2.02E-09 |
| negative regulation of cellular biosynthetic process | 1220 | 8.81 | 29 | 5.27E-09 |
| negative regulation of biosynthetic process | 1240 | 8.95 | 29 | 7.61E-09 |
| negative regulation of transcription, DNA-dependent | 987 | 7.12 | 24 | 9.74E-08 |
| negative regulation of transcription, DNA-dependent | 987 | 7.12 | 24 | 9.74E-08 |
| negative regulation of RNA metabolic process | 1020 | 7.38 | 24 | 1.93E-07 |
| negative regulation of nucleobase-containing compound metabolic process | 1130 | 8.15 | 25 | 3.08E-07 |
| generation of neurons | 1300 | 9.4 | 26 | 1.23E-06 |
| cell morphogenesis involved in differentiation | 827 | 5.96 | 20 | 1.52E-06 |
| regulation of transcription from RNA polymerase II promoter | 1610 | 11.6 | 29 | 1.97E-06 |
| negative regulation of cellular process | 4110 | 29.6 | 52 | 2.53E-06 |
| cell development | 1840 | 13.3 | 31 | 3.45E-06 |
| axonogenesis | 578 | 4.17 | 16 | 3.58E-06 |
| neurogenesis | 1390 | 10 | 26 | 4.10E-06 |
| negative regulation of biological process | 4590 | 33.1 | 55 | 6.19E-06 |
| regulation of cell proliferation | 1430 | 10.3 | 26 | 6.62E-06 |
| positive regulation of metabolic process | 2690 | 19.4 | 38 | 1.24E-05 |
| ER-nucleus signaling pathway | 111 | 0.8 | 7 | 1.51E-05 |
| negative regulation of response to stimulus | 967 | 6.97 | 20 | 1.59E-05 |
| regulation of gene expression | 4480 | 32.3 | 53 | 1.68E-05 |
| neuron projection development | 816 | 5.88 | 18 | 1.92E-05 |
| transcription from RNA polymerase II promoter | 1930 | 13.9 | 30 | 2.48E-05 |
| enzyme linked receptor protein signaling pathway | 1180 | 8.47 | 22 | 2.62E-05 |
| regulation of body fluid levels | 680 | 4.9 | 16 | 2.72E-05 |
| epidermal growth factor receptor signaling pathway | 167 | 1.2 | 8 | 2.73E-05 |
| response to endogenous stimulus | 1360 | 9.83 | 24 | 2.90E-05 |
| neuron differentiation | 1190 | 8.55 | 22 | 3.03E-05 |
| negative regulation of protein metabolic process | 540 | 3.89 | 14 | 3.20E-05 |
| nervous system development | 2190 | 15.8 | 32 | 4.36E-05 |
| negative regulation of signal transduction | 790 | 5.69 | 17 | 4.62E-05 |
| positive regulation of cellular metabolic process | 2530 | 18.2 | 35 | 4.96E-05 |
| blood coagulation | 564 | 4.07 | 14 | 5.13E-05 |
| cell proliferation | 1900 | 13.7 | 29 | 5.20E-05 |
| coagulation | 568 | 4.09 | 14 | 5.54E-05 |
| hemostasis | 570 | 4.11 | 14 | 5.75E-05 |
| cellular response to stress | 1620 | 11.7 | 26 | 6.38E-05 |
| regulation of protein metabolic process | 1820 | 13.2 | 28 | 6.49E-05 |
| response to chemical stimulus | 3830 | 27.6 | 46 | 6.97E-05 |
| gene silencing | 99 | 0.714 | 6 | 7.90E-05 |
| response to organic substance | 2500 | 18 | 34 | 9.69E-05 |
| regulation of cell differentiation | 1290 | 9.29 | 22 | 0.000106 |
| anatomical structure formation involved in morphogenesis | 2090 | 15.1 | 30 | 0.000113 |
| neuron development | 945 | 6.81 | 18 | 0.00013 |
| positive regulation of cell proliferation | 786 | 5.67 | 16 | 0.000151 |
| regulation of protein stability | 114 | 0.822 | 6 | 0.000172 |
| response to radiation | 345 | 2.49 | 10 | 0.000191 |
| transforming growth factor beta receptor signaling pathway | 221 | 1.59 | 8 | 0.000195 |
| reproductive process | 1740 | 12.6 | 26 | 0.000205 |
| reproduction | 1860 | 13.4 | 27 | 0.000222 |
| cytokinesis | 120 | 0.865 | 6 | 0.000228 |
| positive regulation of cell differentiation | 571 | 4.12 | 13 | 0.000229 |
| positive regulation of developmental process | 817 | 5.89 | 16 | 0.000235 |
| regulation of cellular protein metabolic process | 1560 | 11.2 | 24 | 0.000236 |
| positive regulation of cellular process | 4780 | 34.5 | 52 | 0.000251 |
| regulation of kinase activity | 743 | 5.36 | 15 | 0.000272 |
| regulation of cellular metabolic process | 6120 | 44.1 | 62 | 0.000281 |
| regulation of neurogenesis | 444 | 3.2 | 11 | 0.000351 |
| viral reproductive process | 597 | 4.3 | 13 | 0.000351 |
| regulation of transferase activity | 768 | 5.54 | 15 | 0.000386 |
| regulation of protein kinase activity | 698 | 5.03 | 14 | 0.000472 |
| regulation of metabolic process | 6920 | 49.9 | 67 | 0.000482 |
| wound healing | 700 | 5.05 | 14 | 0.000485 |
| negative regulation of cellular protein metabolic process | 463 | 3.34 | 11 | 5.00E-04 |
| negative regulation of cell differentiation | 540 | 3.89 | 12 | 0.000502 |
| response to abiotic stimulus | 876 | 6.31 | 16 | 0.00051 |
| positive regulation of cellular protein metabolic process | 968 | 6.98 | 17 | 0.000526 |
| response to stress | 4150 | 29.9 | 46 | 0.000528 |
| DNA-dependent transcription, initiation | 257 | 1.85 | 8 | 0.000536 |
| axon guidance | 394 | 2.84 | 10 | 0.000546 |
| regulation of nucleobase-containing compound metabolic process | 4540 | 32.7 | 49 | 0.000558 |
| DNA damage checkpoint | 143 | 1.03 | 6 | 0.000583 |
| regulation of developmental process | 1880 | 13.5 | 26 | 0.000656 |
| positive regulation of protein metabolic process | 1080 | 7.81 | 18 | 0.00068 |
| phosphatidylinositol-mediated signaling | 148 | 1.07 | 6 | 0.000699 |
| DNA integrity checkpoint | 152 | 1.1 | 6 | 0.000804 |
| regulation of gene expression, epigenetic | 155 | 1.12 | 6 | 0.00089 |
| regulation of biological quality | 3400 | 24.5 | 39 | 0.000953 |
| response to wounding | 1310 | 9.47 | 20 | 0.000994 |
| regulation of transcription, DNA-dependent | 3770 | 27.2 | 42 | 0.00101 |
| regulation of transcription, DNA-dependent | 3770 | 27.2 | 42 | 0.00101 |
| regulation of transcription, DNA-dependent | 3770 | 27.2 | 42 | 0.00101 |
| transcription initiation from RNA polymerase II promoter | 219 | 1.58 | 7 | 0.00103 |
| regulation of transforming growth factor beta receptor signaling pathway | 107 | 0.771 | 5 | 0.00105 |
| cell division | 507 | 3.65 | 11 | 0.00106 |
| regulation of RNA metabolic process | 3900 | 28.1 | 43 | 0.00107 |
| negative regulation of developmental process | 674 | 4.86 | 13 | 0.00109 |
| negative regulation of apoptotic process | 679 | 4.89 | 13 | 0.00117 |
| negative regulation of apoptotic process | 679 | 4.89 | 13 | 0.00117 |
| regulation of catabolic process | 595 | 4.29 | 12 | 0.00117 |
| response to UV | 112 | 0.807 | 5 | 0.00129 |
| response to ionizing radiation | 112 | 0.807 | 5 | 0.00129 |
| response to nutrient levels | 295 | 2.13 | 8 | 0.00131 |
| positive regulation of protein modification process | 867 | 6.25 | 15 | 0.00134 |
| negative regulation of programmed cell death | 691 | 4.98 | 13 | 0.00137 |
| transmembrane receptor protein tyrosine kinase signaling pathway | 782 | 5.64 | 14 | 0.00142 |
| regulation of DNA metabolic process | 235 | 1.69 | 7 | 0.00155 |
| regulation of cell cycle | 886 | 6.39 | 15 | 0.00166 |
| gliogenesis | 176 | 1.27 | 6 | 0.00171 |

**Table S2e. GO-Molecular functions enrichment analysis of shared-DEmiRNA-gene network**

| **GO Molecular Function** | **Total** | **Expected** | **Hits** | **P value** |
| --- | --- | --- | --- | --- |
| protein kinase binding | 376 | 2.57 | 16 | 4.93E-09 |
| kinase binding | 418 | 2.86 | 16 | 2.20E-08 |
| negative regulation of transcription, DNA-dependent | 987 | 6.75 | 24 | 3.35E-08 |
| chromatin binding | 338 | 2.31 | 13 | 4.92E-07 |
| transcription factor binding | 509 | 3.48 | 15 | 1.78E-06 |
| enzyme binding | 1200 | 8.2 | 23 | 4.41E-06 |
| transcription from RNA polymerase II promoter | 1930 | 13.2 | 30 | 8.05E-06 |
| transcription corepressor activity | 208 | 1.42 | 8 | 8.85E-05 |
| SMAD binding | 68 | 0.465 | 5 | 1.00E-04 |
| protein binding transcription factor activity | 600 | 4.1 | 12 | 0.000788 |
| nucleotide binding | 2470 | 16.9 | 30 | 0.000803 |
| transcription cofactor activity | 552 | 3.77 | 11 | 0.00135 |
| sequence-specific DNA binding | 732 | 5 | 13 | 0.00141 |
| purine ribonucleotide binding | 1890 | 12.9 | 24 | 0.00175 |
| purine nucleotide binding | 1900 | 13 | 24 | 0.00183 |
| DNA binding | 2760 | 18.9 | 31 | 0.00232 |
| ATP binding | 1490 | 10.2 | 20 | 0.00232 |
| adenyl ribonucleotide binding | 1530 | 10.4 | 20 | 0.00312 |
| adenyl nucleotide binding | 1530 | 10.5 | 20 | 0.00321 |
| chaperone binding | 46 | 0.314 | 3 | 0.0038 |
| cation-transporting ATPase activity | 52 | 0.356 | 3 | 0.00537 |
| ATPase activity, coupled to transmembrane movement of ions, phosphorylative mechanism | 54 | 0.369 | 3 | 0.00597 |
| structure-specific DNA binding | 242 | 1.65 | 6 | 0.0063 |
| RNA polymerase II distal enhancer sequence-specific DNA binding transcription factor activity | 110 | 0.752 | 4 | 0.00683 |
| translation regulator activity | 23 | 0.157 | 2 | 0.0107 |
| ATPase activity, coupled to transmembrane movement of ions | 77 | 0.526 | 3 | 0.0157 |
| ion binding | 6140 | 42 | 53 | 0.0166 |
| nucleoside-triphosphatase activity | 888 | 6.07 | 12 | 0.0178 |
| double-stranded DNA binding | 149 | 1.02 | 4 | 0.0191 |
| peptide binding | 159 | 1.09 | 4 | 0.0236 |
| ATPase activity | 417 | 2.85 | 7 | 0.0241 |
| pyrophosphatase activity | 938 | 6.41 | 12 | 0.026 |
| hydrolase activity, acting on acid anhydrides | 944 | 6.45 | 12 | 0.0272 |
| protein complex binding | 339 | 2.32 | 6 | 0.0287 |
| RNA binding | 976 | 6.67 | 12 | 0.034 |
| translation factor activity, nucleic acid binding | 108 | 0.738 | 3 | 0.0378 |
| protein domain specific binding | 560 | 3.83 | 8 | 0.0378 |
| damaged DNA binding | 46 | 0.314 | 2 | 0.0394 |
| collagen binding | 47 | 0.321 | 2 | 0.041 |
| phosphatase binding | 116 | 0.793 | 3 | 0.0451 |
| hydrolase activity, acting on acid anhydrides, catalyzing transmembrane movement of substances | 119 | 0.814 | 3 | 0.0481 |
| ATPase activity, coupled to movement of substances | 121 | 0.827 | 3 | 0.0501 |
| S-adenosylmethionine-dependent methyltransferase activity | 123 | 0.841 | 3 | 0.0521 |
| primary active transmembrane transporter activity | 125 | 0.855 | 3 | 0.0542 |
| growth factor binding | 125 | 0.855 | 3 | 0.0542 |
| cation binding | 4160 | 28.4 | 36 | 0.0599 |
| ATPase activity, coupled | 313 | 2.14 | 5 | 0.0634 |
| cytoskeletal protein binding | 738 | 5.05 | 9 | 0.0653 |
| hormone binding | 64 | 0.438 | 2 | 0.071 |
| receptor binding | 1590 | 10.9 | 16 | 0.0743 |
| GTPase activity | 241 | 1.65 | 4 | 0.0832 |
| actin filament binding | 71 | 0.485 | 2 | 0.0849 |
| positive regulation of transcription, DNA-dependent | 1260 | 8.6 | 13 | 0.0867 |
| RNA polymerase II transcription cofactor activity | 73 | 0.499 | 2 | 0.089 |
| protein phosphatase binding | 74 | 0.506 | 2 | 0.0911 |
| insulin-like growth factor receptor binding | 14 | 0.0957 | 1 | 0.0916 |
| protein C-terminus binding | 160 | 1.09 | 3 | 0.0967 |
| low-density lipoprotein particle binding | 15 | 0.103 | 1 | 0.0978 |
| structural constituent of cytoskeleton | 78 | 0.533 | 2 | 0.0995 |
| extracellular matrix structural constituent | 80 | 0.547 | 2 | 0.104 |
| telomeric DNA binding | 17 | 0.116 | 1 | 0.11 |
| actin binding | 373 | 2.55 | 5 | 0.112 |
| integrin binding | 86 | 0.588 | 2 | 0.117 |
| protein N-terminus binding | 86 | 0.588 | 2 | 0.117 |
| transmembrane receptor protein tyrosine kinase activity | 88 | 0.602 | 2 | 0.122 |
| magnesium ion binding | 188 | 1.29 | 3 | 0.138 |
| nucleotide kinase activity | 23 | 0.157 | 1 | 0.146 |
| ADP binding | 23 | 0.157 | 1 | 0.146 |
| mRNA binding | 99 | 0.677 | 2 | 0.147 |
| neutral amino acid transmembrane transporter activity | 26 | 0.178 | 1 | 0.163 |
| lipoprotein particle binding | 26 | 0.178 | 1 | 0.163 |
| transcription coactivator activity | 312 | 2.13 | 4 | 0.165 |
| identical protein binding | 910 | 6.22 | 9 | 0.169 |
| calcium ion binding | 673 | 4.6 | 7 | 0.177 |
| RNA helicase activity | 30 | 0.205 | 1 | 0.186 |
| protein tyrosine kinase activity | 222 | 1.52 | 3 | 0.194 |
| methyltransferase activity | 222 | 1.52 | 3 | 0.194 |
| protein tyrosine phosphatase activity | 119 | 0.814 | 2 | 0.196 |
| SH2 domain binding | 33 | 0.226 | 1 | 0.203 |
| metalloendopeptidase activity | 123 | 0.841 | 2 | 0.206 |
| Ras GTPase binding | 125 | 0.855 | 2 | 0.211 |
| transmembrane receptor protein kinase activity | 125 | 0.855 | 2 | 0.211 |
| transferase activity, transferring one-carbon groups | 233 | 1.59 | 3 | 0.214 |
| deaminase activity | 35 | 0.239 | 1 | 0.214 |
| calcium ion transmembrane transporter activity | 130 | 0.889 | 2 | 0.223 |
| carboxy-lyase activity | 38 | 0.26 | 1 | 0.23 |
| Rho GTPase activator activity | 39 | 0.267 | 1 | 0.235 |
| nucleotidyltransferase activity | 135 | 0.923 | 2 | 0.236 |
| protein binding, bridging | 135 | 0.923 | 2 | 0.236 |
| small GTPase binding | 138 | 0.943 | 2 | 0.243 |
| endonuclease activity, active with either ribo- or deoxyribonucleic acids and producing 5'-phosphomonoesters | 41 | 0.28 | 1 | 0.245 |
| GTP binding | 371 | 2.54 | 4 | 0.249 |
| hydrolase activity, acting on carbon-nitrogen (but not peptide) bonds, in cyclic amidines | 42 | 0.287 | 1 | 0.251 |
| L-amino acid transmembrane transporter activity | 43 | 0.294 | 1 | 0.256 |
| RNA polymerase activity | 44 | 0.301 | 1 | 0.261 |
| phosphotransferase activity, phosphate group as acceptor | 44 | 0.301 | 1 | 0.261 |
| binding, bridging | 148 | 1.01 | 2 | 0.269 |
| non-membrane spanning protein tyrosine kinase activity | 46 | 0.314 | 1 | 0.271 |
| GTPase binding | 150 | 1.03 | 2 | 0.274 |
| double-stranded RNA binding | 49 | 0.335 | 1 | 0.286 |

**Table S2f : GO Cellular Component enrichment analysis of shared-DEmiRNA-gene network**

| **GO Cellular Component** | **Total** | **Expected** | **Hits** | **P value** |
| --- | --- | --- | --- | --- |
| cytosol | 2660 | 16.3 | 37 | 4.70E-07 |
| nucleoplasm | 1820 | 11.2 | 28 | 2.96E-06 |
| nuclear lumen | 2690 | 16.5 | 35 | 5.22E-06 |
| organelle lumen | 3380 | 20.7 | 40 | 8.75E-06 |
| nucleoplasm part | 910 | 5.57 | 18 | 9.20E-06 |
| macromolecular complex | 4800 | 29.3 | 50 | 1.19E-05 |
| membrane-enclosed lumen | 3440 | 21.1 | 40 | 1.35E-05 |
| transcription factor complex | 303 | 1.85 | 10 | 1.65E-05 |
| membrane-bounded vesicle | 1100 | 6.72 | 19 | 3.32E-05 |
| nuclear chromatin | 159 | 0.973 | 7 | 5.44E-05 |
| nuclear part | 3330 | 20.4 | 37 | 9.38E-05 |
| vesicle | 1210 | 7.4 | 19 | 0.000119 |
| nuclear chromosome | 320 | 1.96 | 9 | 0.000152 |
| nuclear chromosome part | 273 | 1.67 | 8 | 0.000271 |
| cytoplasmic membrane-bounded vesicle | 1020 | 6.26 | 16 | 0.000465 |
| nucleus | 7600 | 46.5 | 63 | 0.000734 |
| chromatin | 326 | 1.99 | 8 | 0.000875 |
| cytoplasmic vesicle | 1110 | 6.79 | 16 | 0.00111 |
| protein complex | 4050 | 24.8 | 39 | 0.00119 |
| PML body | 77 | 0.471 | 4 | 0.00127 |
| nuclear matrix | 87 | 0.532 | 4 | 0.00199 |
| perinuclear region of cytoplasm | 475 | 2.91 | 9 | 0.00253 |
| clathrin-coated vesicle | 224 | 1.37 | 6 | 0.00254 |
| chromosomal part | 670 | 4.1 | 11 | 0.00263 |
| ribonucleoprotein complex | 681 | 4.17 | 11 | 0.00297 |
| replication fork | 50 | 0.306 | 3 | 0.00354 |
| spindle | 261 | 1.6 | 6 | 0.00536 |
| endocytic vesicle | 187 | 1.14 | 5 | 0.00584 |
| extracellular region part | 1320 | 8.08 | 16 | 0.00629 |
| trans-Golgi network | 120 | 0.734 | 4 | 0.00631 |
| coated vesicle | 283 | 1.73 | 6 | 0.00786 |
| extracellular matrix part | 204 | 1.25 | 5 | 0.00835 |
| chromosome | 784 | 4.8 | 11 | 0.00842 |
| immunological synapse | 23 | 0.141 | 2 | 0.00863 |
| Golgi-associated vesicle | 70 | 0.428 | 3 | 0.00905 |
| nuclear body | 295 | 1.81 | 6 | 0.00953 |
| integrin complex | 32 | 0.196 | 2 | 0.0163 |
| cytoplasmic vesicle part | 462 | 2.83 | 7 | 0.0233 |
| basement membrane | 100 | 0.612 | 3 | 0.0235 |
| neuron projection | 685 | 4.19 | 9 | 0.0244 |
| secretory granule | 276 | 1.69 | 5 | 0.0273 |
| non-membrane-bounded organelle | 3940 | 24.1 | 33 | 0.0281 |
| intracellular non-membrane-bounded organelle | 3940 | 24.1 | 33 | 0.0281 |
| membrane raft | 189 | 1.16 | 4 | 0.0288 |
| cell leading edge | 283 | 1.73 | 5 | 0.0299 |
| nuclear envelope | 387 | 2.37 | 6 | 0.0315 |
| cell cortex | 195 | 1.19 | 4 | 0.0318 |
| proteinaceous extracellular matrix | 398 | 2.44 | 6 | 0.0354 |
| cytoplasmic vesicle membrane | 403 | 2.47 | 6 | 0.0373 |
| vesicle membrane | 417 | 2.55 | 6 | 0.0429 |
| integral to plasma membrane | 1270 | 7.77 | 13 | 0.0459 |
| nucleolus | 652 | 3.99 | 8 | 0.0465 |
| cortical cytoskeleton | 60 | 0.367 | 2 | 0.0522 |
| intrinsic to plasma membrane | 1320 | 8.1 | 13 | 0.0599 |
| coated vesicle membrane | 162 | 0.991 | 3 | 0.0772 |
| DNA-directed RNA polymerase II, core complex | 14 | 0.0857 | 1 | 0.0824 |
| axon | 269 | 1.65 | 4 | 0.0831 |
| endoplasmic reticulum lumen | 175 | 1.07 | 3 | 0.0922 |
| cell body | 290 | 1.77 | 4 | 0.102 |
| membrane coat | 89 | 0.545 | 2 | 0.103 |
| coated membrane | 89 | 0.545 | 2 | 0.103 |
| endomembrane system | 2160 | 13.2 | 18 | 0.105 |
| plasma membrane part | 2320 | 14.2 | 19 | 0.109 |
| receptor complex | 189 | 1.16 | 3 | 0.11 |
| DNA-directed RNA polymerase II, holoenzyme | 93 | 0.569 | 2 | 0.111 |
| cell cortex part | 97 | 0.594 | 2 | 0.119 |
| transcription factor TFIID complex | 21 | 0.128 | 1 | 0.121 |
| chromosome, centromeric region | 198 | 1.21 | 3 | 0.121 |
| actin cytoskeleton | 430 | 2.63 | 5 | 0.124 |
| DNA-directed RNA polymerase complex | 105 | 0.642 | 2 | 0.135 |
| nuclear DNA-directed RNA polymerase complex | 105 | 0.642 | 2 | 0.135 |
| extracellular matrix | 570 | 3.49 | 6 | 0.137 |
| U12-type spliceosomal complex | 24 | 0.147 | 1 | 0.137 |
| RNA polymerase complex | 106 | 0.649 | 2 | 0.137 |
| trans-Golgi network transport vesicle | 26 | 0.159 | 1 | 0.148 |
| early endosome | 217 | 1.33 | 3 | 0.148 |
| cell projection | 1410 | 8.62 | 12 | 0.151 |
| dendrite | 342 | 2.09 | 4 | 0.158 |
| nuclear replication fork | 28 | 0.171 | 1 | 0.158 |
| extracellular space | 901 | 5.51 | 8 | 0.186 |
| ruffle | 135 | 0.826 | 2 | 0.2 |
| intrinsic to organelle membrane | 255 | 1.56 | 3 | 0.205 |
| small nuclear ribonucleoprotein complex | 40 | 0.245 | 1 | 0.218 |
| intracellular organelle part | 8620 | 52.7 | 57 | 0.226 |
| cell-substrate junction | 147 | 0.899 | 2 | 0.227 |
| endosome | 683 | 4.18 | 6 | 0.24 |
| vesicle coat | 45 | 0.275 | 1 | 0.242 |
| synapse | 558 | 3.41 | 5 | 0.256 |
| spliceosomal complex | 161 | 0.985 | 2 | 0.259 |
| basolateral plasma membrane | 162 | 0.991 | 2 | 0.261 |
| histone deacetylase complex | 53 | 0.324 | 1 | 0.278 |
| late endosome | 175 | 1.07 | 2 | 0.29 |
| organelle part | 8790 | 53.8 | 57 | 0.295 |
| endoplasmic reticulum | 1660 | 10.2 | 12 | 0.315 |
| endoplasmic reticulum part | 1060 | 6.46 | 8 | 0.318 |
| external side of plasma membrane | 204 | 1.25 | 2 | 0.356 |
| condensed nuclear chromosome | 73 | 0.447 | 1 | 0.362 |
| microtubule | 352 | 2.15 | 3 | 0.365 |
| myosin complex | 75 | 0.459 | 1 | 0.37 |
| microtubule cytoskeleton | 1120 | 6.84 | 8 | 0.377 |

**Table S3a. Shared-DEmiRNA-TF network-interactions**

| **Node** | **Degree** | **Betweenness** |
| --- | --- | --- |
| hsa-mir-29a | 5 | 34.02857 |
| has-mir-155 | 5 | 27.14048 |
| hsa-mir-223 | 4 | 35.15238 |
| hsa-mir-143 | 4 | 19.0631 |
| STAT3 | 4 | 17.60714 |
| EZH2 | 3 | 11.78571 |
| HIF1A | 3 | 6.616667 |
| hsa-mir-204 | 3 | 5.371429 |
| CEBPA | 2 | 15.53333 |
| RUNX1 | 2 | 15 |
| RELA | 2 | 11.55952 |
| CEBPB | 2 | 10.05952 |
| MYC | 2 | 5.708333 |
| hsa-mir-30a | 2 | 3.244048 |
| HDAC4 | 2 | 3.227381 |
| MITF | 2 | 2.902381 |
| hsa-mir-484 | 1 | 0 |

**Table S3b. KEGG pathway enrichment analysis of Shared-DEmiRNA-TF network-interactions**

| **KEGG Pathway** | **Total** | **Expected** | **Hits** | **P value** |
| --- | --- | --- | --- | --- |
| Acute myeloid leukemia | 57 | 0.11 | 5 | 2.72E-08 |
| Pathways in cancer | 310 | 0.596 | 6 | 5.71E-06 |
| Chronic myeloid leukemia | 73 | 0.14 | 3 | 0.000286 |
| Epstein-Barr virus infection | 91 | 0.175 | 3 | 0.000549 |
| Transcriptional misregulation in cancer | 19 | 0.0365 | 2 | 0.000553 |
| Adipocytokine signaling pathway | 63 | 0.121 | 2 | 0.00604 |
| Pancreatic cancer | 69 | 0.133 | 2 | 0.00721 |
| Small cell lung cancer | 80 | 0.154 | 2 | 0.00961 |
| Toxoplasmosis | 93 | 0.179 | 2 | 0.0128 |
| Jak-STAT signaling pathway | 99 | 0.19 | 2 | 0.0145 |
| Measles | 102 | 0.196 | 2 | 0.0153 |
| Cytosolic DNA-sensing pathway | 20 | 0.0385 | 1 | 0.0378 |
| Tuberculosis | 174 | 0.335 | 2 | 0.0417 |
| Chemokine signaling pathway | 189 | 0.363 | 2 | 0.0484 |
| Thyroid cancer | 28 | 0.0538 | 1 | 0.0526 |
| Epithelial cell signaling in Helicobacter pylori infection | 37 | 0.0712 | 1 | 0.069 |
| Legionellosis | 40 | 0.0769 | 1 | 0.0744 |
| Cocaine addiction | 43 | 0.0827 | 1 | 0.0798 |
| Endometrial cancer | 44 | 0.0846 | 1 | 0.0816 |
| mTOR signaling pathway | 45 | 0.0865 | 1 | 0.0834 |
| Amoebiasis | 46 | 0.0885 | 1 | 0.0851 |
| Shigellosis | 47 | 0.0904 | 1 | 0.0869 |
| MAPK signaling pathway | 265 | 0.51 | 2 | 0.0884 |
| NOD-like receptor signaling pathway | 49 | 0.0942 | 1 | 0.0905 |
| RIG-I-like receptor signaling pathway | 49 | 0.0942 | 1 | 0.0905 |
| Colorectal cancer | 49 | 0.0942 | 1 | 0.0905 |
| Leishmaniasis | 51 | 0.0981 | 1 | 0.094 |
| Pertussis | 52 | 0.1 | 1 | 0.0957 |
| Renal cell carcinoma | 60 | 0.115 | 1 | 0.11 |
| Salmonella infection | 72 | 0.138 | 1 | 0.13 |
| B cell receptor signaling pathway | 75 | 0.144 | 1 | 0.135 |
| Apoptosis | 83 | 0.16 | 1 | 0.149 |
| TGF-beta signaling pathway | 84 | 0.162 | 1 | 0.151 |
| ErbB signaling pathway | 87 | 0.167 | 1 | 0.156 |
| Prostate cancer | 87 | 0.167 | 1 | 0.156 |
| Chagas disease (American trypanosomiasis) | 89 | 0.171 | 1 | 0.159 |
| Toll-like receptor signaling pathway | 97 | 0.187 | 1 | 0.172 |
| T cell receptor signaling pathway | 98 | 0.188 | 1 | 0.174 |
| Hepatitis C | 100 | 0.192 | 1 | 0.177 |
| Melanogenesis | 101 | 0.194 | 1 | 0.178 |
| Herpes simplex infection | 103 | 0.198 | 1 | 0.182 |
| Influenza A | 107 | 0.206 | 1 | 0.188 |
| Osteoclast differentiation | 119 | 0.229 | 1 | 0.207 |
| Neurotrophin signaling pathway | 123 | 0.237 | 1 | 0.213 |
| Cell cycle | 124 | 0.238 | 1 | 0.215 |
| Wnt signaling pathway | 144 | 0.277 | 1 | 0.245 |
| Alcoholism | 166 | 0.319 | 1 | 0.278 |
| HTLV-I infection | 199 | 0.383 | 1 | 0.324 |

**Table S3c. Reactome pathway enrichment analysis of Shared-DEmiRNA-TF network-interactions**

| **Reactome Pathway** | **Total** | **Expected** | **Hits** | **P value** |
| --- | --- | --- | --- | --- |
| Cellular responses to stress | 256 | 0.266 | 4 | 6.47E-05 |
| Cellular Senescence | 143 | 0.148 | 3 | 0.000306 |
| NOTCH1 Intracellular Domain Regulates Transcription | 40 | 0.0415 | 2 | 0.000706 |
| Transcriptional regulation of white adipocyte differentiation | 57 | 0.0591 | 2 | 0.00143 |
| Signaling by NOTCH1 | 60 | 0.0622 | 2 | 0.00159 |
| Senescence-Associated Secretory Phenotype (SASP) | 72 | 0.0747 | 2 | 0.00228 |
| Signaling by NOTCH | 86 | 0.0892 | 2 | 0.00323 |
| DEx/H-box helicases activate type I IFN and inflammatory cytokines production | 7 | 0.00726 | 1 | 0.00724 |
| binding of TCF/LEF:CTNNB1 to target gene promoters | 7 | 0.00726 | 1 | 0.00724 |
| IkBA variant leads to EDA-ID | 7 | 0.00726 | 1 | 0.00724 |
| Developmental Biology | 438 | 0.454 | 3 | 0.0078 |
| IKBKG deficiency causes anhidrotic ectodermal dysplasia with immunodeficiency (EDA-ID) (via TLR) | 8 | 0.0083 | 1 | 0.00827 |
| Regulation of gene expression by Hypoxia-inducible Factor | 9 | 0.00933 | 1 | 0.0093 |
| Interleukin-6 signaling | 10 | 0.0104 | 1 | 0.0103 |
| Regulated proteolysis of p75NTR | 10 | 0.0104 | 1 | 0.0103 |
| RIP-mediated NFkB activation via ZBP1 | 11 | 0.0114 | 1 | 0.0114 |
| ZBP1(DAI) mediated induction of type I IFNs | 13 | 0.0135 | 1 | 0.0134 |
| NF-kB is activated and signals survival | 13 | 0.0135 | 1 | 0.0134 |
| Signaling by FGFR1 fusion mutants | 16 | 0.0166 | 1 | 0.0165 |
| p75NTR signals via NF-kB | 16 | 0.0166 | 1 | 0.0165 |
| TRAF6 mediated NF-kB activation | 16 | 0.0166 | 1 | 0.0165 |
| Oxygen-dependent proline hydroxylation of Hypoxia-inducible Factor Alpha | 18 | 0.0187 | 1 | 0.0185 |
| Signaling by Leptin | 21 | 0.0218 | 1 | 0.0216 |
| POU5F1 (OCT4), SOX2, NANOG activate genes related to proliferation | 21 | 0.0218 | 1 | 0.0216 |
| CD209 (DC-SIGN) signaling | 21 | 0.0218 | 1 | 0.0216 |
| Diseases of signal transduction | 235 | 0.244 | 2 | 0.0226 |
| TAK1 activates NFkB by phosphorylation and activation of IKKs complex | 22 | 0.0228 | 1 | 0.0226 |
| Growth hormone receptor signaling | 23 | 0.0239 | 1 | 0.0236 |
| Diseases of Immune System | 24 | 0.0249 | 1 | 0.0246 |
| Diseases associated with the TLR signaling cascade | 24 | 0.0249 | 1 | 0.0246 |
| Disease | 669 | 0.694 | 3 | 0.025 |
| Regulation of Hypoxia-inducible Factor (HIF) by oxygen | 25 | 0.0259 | 1 | 0.0257 |
| Cellular response to hypoxia | 25 | 0.0259 | 1 | 0.0257 |
| FGFR1 mutant receptor activation | 27 | 0.028 | 1 | 0.0277 |
| SMAD2/SMAD3:SMAD4 heterotrimer regulates transcription | 28 | 0.029 | 1 | 0.0287 |
| FCERI mediated NF-kB activation | 29 | 0.0301 | 1 | 0.0297 |
| Signalling by NGF | 273 | 0.283 | 2 | 0.0299 |
| Transcriptional regulation of pluripotent stem cells | 30 | 0.0311 | 1 | 0.0307 |
| Signaling by FGFR1 in disease | 33 | 0.0342 | 1 | 0.0337 |
| Signaling by FGFR1 mutants | 33 | 0.0342 | 1 | 0.0337 |
| Signaling by FGFR in disease | 42 | 0.0436 | 1 | 0.0428 |
| PRC2 methylates histones and DNA | 42 | 0.0436 | 1 | 0.0428 |
| Transcriptional activity of SMAD2/SMAD3:SMAD4 heterotrimer | 43 | 0.0446 | 1 | 0.0438 |
| formation of the beta-catenin:TCF transactivating complex | 44 | 0.0456 | 1 | 0.0448 |
| Signaling by NOTCH1 PEST Domain Mutants in Cancer | 46 | 0.0477 | 1 | 0.0468 |
| Signaling by NOTCH1 in Cancer | 46 | 0.0477 | 1 | 0.0468 |
| Constitutive Signaling by NOTCH1 PEST Domain Mutants | 46 | 0.0477 | 1 | 0.0468 |
| Signaling by NOTCH1 HD+PEST Domain Mutants in Cancer | 46 | 0.0477 | 1 | 0.0468 |
| Constitutive Signaling by NOTCH1 HD+PEST Domain Mutants | 46 | 0.0477 | 1 | 0.0468 |
| Downstream TCR signaling | 47 | 0.0487 | 1 | 0.0478 |
| PKMTs methylate histone lysines | 50 | 0.0519 | 1 | 0.0507 |
| TRAF6 Mediated Induction of proinflammatory cytokines | 56 | 0.0581 | 1 | 0.0567 |
| Cytosolic sensors of pathogen-associated DNA | 60 | 0.0622 | 1 | 0.0606 |
| TCR signaling | 64 | 0.0664 | 1 | 0.0645 |
| RIG-I/MDA5 mediated induction of IFN-alpha/beta pathways | 65 | 0.0674 | 1 | 0.0655 |
| Activation of NF-kappaB in B cells | 66 | 0.0685 | 1 | 0.0665 |
| Toll Like Receptor 10 (TLR10) Cascade | 67 | 0.0695 | 1 | 0.0675 |
| Toll Like Receptor 5 (TLR5) Cascade | 67 | 0.0695 | 1 | 0.0675 |
| MyD88 cascade initiated on plasma membrane | 67 | 0.0695 | 1 | 0.0675 |
| TRAF6 mediated induction of NFkB and MAP kinases upon TLR7/8 or 9 activation | 68 | 0.0705 | 1 | 0.0685 |
| Toll Like Receptor 7/8 (TLR7/8) Cascade | 69 | 0.0716 | 1 | 0.0694 |
| MyD88 dependent cascade initiated on endosome | 69 | 0.0716 | 1 | 0.0694 |
| Toll Like Receptor 9 (TLR9) Cascade | 71 | 0.0736 | 1 | 0.0714 |
| Signaling by TGF-beta Receptor Complex | 72 | 0.0747 | 1 | 0.0724 |
| MyD88:Mal cascade initiated on plasma membrane | 74 | 0.0768 | 1 | 0.0743 |
| Toll Like Receptor TLR1:TLR2 Cascade | 74 | 0.0768 | 1 | 0.0743 |
| Toll Like Receptor TLR6:TLR2 Cascade | 74 | 0.0768 | 1 | 0.0743 |
| Toll Like Receptor 2 (TLR2) Cascade | 74 | 0.0768 | 1 | 0.0743 |
| TRIF-mediated TLR3/TLR4 signaling | 77 | 0.0799 | 1 | 0.0772 |
| MyD88-independent TLR3/TLR4 cascade | 78 | 0.0809 | 1 | 0.0782 |
| Toll Like Receptor 3 (TLR3) Cascade | 78 | 0.0809 | 1 | 0.0782 |
| p75 NTR receptor-mediated signalling | 86 | 0.0892 | 1 | 0.0859 |
| Oxidative Stress Induced Senescence | 88 | 0.0913 | 1 | 0.0878 |
| Activated TLR4 signalling | 93 | 0.0965 | 1 | 0.0926 |
| Epigenetic regulation of gene expression | 94 | 0.0975 | 1 | 0.0936 |
| Toll Like Receptor 4 (TLR4) Cascade | 96 | 0.0996 | 1 | 0.0955 |
| CLEC7A (Dectin-1) signaling | 99 | 0.103 | 1 | 0.0983 |
| Signaling by Interleukins | 107 | 0.111 | 1 | 0.106 |
| Toll-Like Receptors Cascades | 111 | 0.115 | 1 | 0.11 |
| C-type lectin receptors (CLRs) | 123 | 0.128 | 1 | 0.121 |
| Signaling by SCF-KIT | 133 | 0.138 | 1 | 0.13 |
| Signal Transduction | 2070 | 2.15 | 4 | 0.135 |
| Downstream signal transduction | 151 | 0.157 | 1 | 0.147 |
| Downstream signaling events of B Cell Receptor (BCR) | 164 | 0.17 | 1 | 0.158 |
| Fc epsilon receptor (FCERI) signaling | 169 | 0.175 | 1 | 0.163 |
| TCF dependent signaling in response to WNT | 173 | 0.179 | 1 | 0.166 |
| Signaling by PDGF | 177 | 0.184 | 1 | 0.17 |
| NGF signalling via TRKA from the plasma membrane | 189 | 0.196 | 1 | 0.18 |
| Generic Transcription Pathway | 189 | 0.196 | 1 | 0.18 |
| Signaling by the B Cell Receptor (BCR) | 190 | 0.197 | 1 | 0.181 |
| Chromatin modifying enzymes | 216 | 0.224 | 1 | 0.204 |
| Chromatin organization | 216 | 0.224 | 1 | 0.204 |
| Gene Expression | 851 | 0.883 | 2 | 0.218 |
| Signaling by Wnt | 245 | 0.254 | 1 | 0.228 |
| Immune System | 942 | 0.977 | 2 | 0.254 |
| Cytokine Signaling in Immune system | 284 | 0.295 | 1 | 0.26 |
| Adaptive Immune System | 430 | 0.446 | 1 | 0.369 |
| Innate Immune System | 569 | 0.59 | 1 | 0.46 |

**Table S3d. GO-BP Pathways enrichement analysis in Shared-DEmiRNA-TF network-interactions**

| **GO Biological Pathway** | **Total** | **Expected** | **Hits** | **P value** |
| --- | --- | --- | --- | --- |
| regulation of cell differentiation | 1290 | 0.902 | 10 | 3.46E-11 |
| regulation of developmental process | 1880 | 1.31 | 10 | 1.51E-09 |
| cell proliferation | 1900 | 1.33 | 10 | 1.72E-09 |
| transcription from RNA polymerase II promoter | 1930 | 1.35 | 10 | 1.97E-09 |
| positive regulation of transcription from RNA polymerase II promoter | 800 | 0.56 | 8 | 3.80E-09 |
| regulation of multicellular organismal process | 2480 | 1.74 | 10 | 2.44E-08 |
| regulation of transcription from RNA polymerase II promoter | 1610 | 1.13 | 9 | 2.55E-08 |
| positive regulation of transcription, DNA-dependent | 1260 | 0.88 | 8 | 1.35E-07 |
| positive regulation of transcription, DNA-dependent | 1260 | 0.88 | 8 | 1.35E-07 |
| positive regulation of RNA metabolic process | 1330 | 0.933 | 8 | 2.13E-07 |
| regulation of cell proliferation | 1430 | 0.999 | 8 | 3.66E-07 |
| organ development | 3290 | 2.3 | 10 | 4.17E-07 |
| positive regulation of nucleobase-containing compound metabolic process | 1490 | 1.04 | 8 | 5.10E-07 |
| inflammatory response | 569 | 0.398 | 6 | 7.12E-07 |
| negative regulation of transcription, DNA-dependent | 987 | 0.691 | 7 | 7.33E-07 |
| negative regulation of transcription, DNA-dependent | 987 | 0.691 | 7 | 7.33E-07 |
| myeloid cell differentiation | 296 | 0.207 | 5 | 8.54E-07 |
| negative regulation of RNA metabolic process | 1020 | 0.717 | 7 | 9.43E-07 |
| hemopoiesis | 640 | 0.448 | 6 | 1.42E-06 |
| regulation of transcription, DNA-dependent | 3770 | 2.64 | 10 | 1.63E-06 |
| regulation of transcription, DNA-dependent | 3770 | 2.64 | 10 | 1.63E-06 |
| regulation of transcription, DNA-dependent | 3770 | 2.64 | 10 | 1.63E-06 |
| negative regulation of nucleobase-containing compound metabolic process | 1130 | 0.792 | 7 | 1.85E-06 |
| negative regulation of developmental process | 674 | 0.472 | 6 | 1.92E-06 |
| hematopoietic or lymphoid organ development | 679 | 0.475 | 6 | 2.01E-06 |
| regulation of myeloid cell differentiation | 145 | 0.101 | 4 | 2.04E-06 |
| regulation of RNA metabolic process | 3900 | 2.73 | 10 | 2.30E-06 |
| myeloid leukocyte differentiation | 153 | 0.107 | 4 | 2.53E-06 |
| immune system development | 722 | 0.505 | 6 | 2.88E-06 |
| negative regulation of cellular biosynthetic process | 1220 | 0.855 | 7 | 3.14E-06 |
| negative regulation of biosynthetic process | 1240 | 0.869 | 7 | 3.50E-06 |
| negative regulation of cellular process | 4110 | 2.87 | 10 | 3.81E-06 |
| leukocyte differentiation | 404 | 0.283 | 5 | 3.95E-06 |
| positive regulation of developmental process | 817 | 0.572 | 6 | 5.91E-06 |
| regulation of gene expression | 4480 | 3.14 | 10 | 9.18E-06 |
| regulation of nucleobase-containing compound metabolic process | 4540 | 3.18 | 10 | 1.04E-05 |
| negative regulation of biological process | 4590 | 3.21 | 10 | 1.17E-05 |
| organ morphogenesis | 966 | 0.676 | 6 | 1.56E-05 |
| negative regulation of cell differentiation | 540 | 0.378 | 5 | 1.63E-05 |
| transcription, DNA-dependent | 4830 | 3.38 | 10 | 1.95E-05 |
| transcription, DNA-dependent | 4830 | 3.38 | 10 | 1.95E-05 |
| positive regulation of cell differentiation | 571 | 0.4 | 5 | 2.14E-05 |
| RNA biosynthetic process | 4930 | 3.45 | 10 | 2.38E-05 |
| system development | 4950 | 3.47 | 10 | 2.49E-05 |
| negative regulation of cellular metabolic process | 1660 | 1.16 | 7 | 2.50E-05 |
| tissue development | 1680 | 1.18 | 7 | 2.72E-05 |
| positive regulation of cellular metabolic process | 2530 | 1.77 | 8 | 3.04E-05 |
| negative regulation of metabolic process | 1820 | 1.28 | 7 | 4.58E-05 |
| positive regulation of metabolic process | 2690 | 1.88 | 8 | 4.92E-05 |
| negative regulation of apoptotic process | 679 | 0.475 | 5 | 4.93E-05 |
| negative regulation of apoptotic process | 679 | 0.475 | 5 | 4.93E-05 |
| cell development | 1840 | 1.29 | 7 | 4.96E-05 |
| immune system process | 2720 | 1.9 | 8 | 5.28E-05 |
| negative regulation of programmed cell death | 691 | 0.484 | 5 | 5.36E-05 |
| response to drug | 344 | 0.241 | 4 | 6.18E-05 |
| acute inflammatory response | 118 | 0.0826 | 3 | 6.32E-05 |
| cytokine-mediated signaling pathway | 374 | 0.262 | 4 | 8.56E-05 |
| cellular nitrogen compound biosynthetic process | 5610 | 3.92 | 10 | 8.62E-05 |
| generation of neurons | 1300 | 0.913 | 6 | 8.69E-05 |
| response to wounding | 1310 | 0.919 | 6 | 9.04E-05 |
| multicellular organismal development | 5720 | 4 | 10 | 0.000105 |
| neurogenesis | 1390 | 0.974 | 6 | 0.000125 |
| anatomical structure development | 5830 | 4.08 | 10 | 0.000128 |
| nervous system development | 2190 | 1.53 | 7 | 0.000155 |
| RNA metabolic process | 6010 | 4.21 | 10 | 0.000174 |
| skeletal system development | 459 | 0.321 | 4 | 0.000189 |
| defense response | 1510 | 1.06 | 6 | 0.000198 |
| gliogenesis | 176 | 0.123 | 3 | 0.000207 |
| regulation of cellular metabolic process | 6120 | 4.28 | 10 | 0.000208 |
| Notch signaling pathway | 177 | 0.124 | 3 | 0.00021 |
| sensory organ development | 485 | 0.339 | 4 | 0.000234 |
| response to organic substance | 2500 | 1.75 | 7 | 0.000364 |
| positive regulation of cellular process | 4780 | 3.34 | 9 | 0.000365 |
| negative regulation of transcription from RNA polymerase II promoter | 552 | 0.386 | 4 | 0.000384 |
| macromolecule biosynthetic process | 6540 | 4.58 | 10 | 0.000405 |
| macromolecule biosynthetic process | 6540 | 4.58 | 10 | 0.000405 |
| response to light stimulus | 229 | 0.16 | 3 | 0.000449 |
| generation of precursor metabolites and energy | 603 | 0.422 | 4 | 0.000538 |
| response to steroid hormone stimulus | 267 | 0.187 | 3 | 0.000703 |
| regulation of immune system process | 1190 | 0.835 | 5 | 0.00071 |
| regulation of metabolic process | 6920 | 4.84 | 10 | 0.00071 |
| intracellular receptor mediated signaling pathway | 270 | 0.189 | 3 | 0.000726 |
| negative regulation of myeloid cell differentiation | 60 | 0.042 | 2 | 0.000764 |
| cellular carbohydrate catabolic process | 62 | 0.0434 | 2 | 0.000815 |
| epidermis development | 319 | 0.223 | 3 | 0.00118 |
| positive regulation of biological process | 5500 | 3.85 | 9 | 0.0012 |
| response to stress | 4150 | 2.91 | 8 | 0.00125 |
| response to endogenous stimulus | 1360 | 0.955 | 5 | 0.00132 |
| peripheral nervous system development | 81 | 0.0567 | 2 | 0.00139 |
| central nervous system development | 784 | 0.549 | 4 | 0.00145 |
| positive regulation of cell proliferation | 786 | 0.55 | 4 | 0.00146 |
| response to radiation | 345 | 0.241 | 3 | 0.00148 |
| negative regulation of signal transduction | 790 | 0.553 | 4 | 0.00149 |
| positive regulation of angiogenesis | 94 | 0.0658 | 2 | 0.00186 |
| regulation of apoptotic process | 1540 | 1.07 | 5 | 0.00225 |
| regulation of cytokine biosynthetic process | 105 | 0.0735 | 2 | 0.00232 |
| cellular biosynthetic process | 7800 | 5.46 | 10 | 0.00236 |
| regulation of programmed cell death | 1550 | 1.09 | 5 | 0.00237 |
| chromatin remodeling | 111 | 0.0777 | 2 | 0.00258 |
| response to UV | 112 | 0.0784 | 2 | 0.00263 |

**Table S3e. GO-Molecular Functions enrichement analysis in Shared-DEmiRNA-TF network-interactions**

| **Molecular Functions** | **Total** | **Expected** | **Hits** | **P value** |
| --- | --- | --- | --- | --- |
| transcription factor binding | 509 | 0.351 | 8 | 9.34E-11 |
| transcription from RNA polymerase II promoter | 1930 | 1.33 | 10 | 1.72E-09 |
| RNA polymerase II distal enhancer sequence-specific DNA binding transcription factor activity | 110 | 0.076 | 5 | 5.64E-09 |
| DNA binding | 2760 | 1.9 | 10 | 6.20E-08 |
| sequence-specific DNA binding | 732 | 0.505 | 7 | 8.61E-08 |
| positive regulation of transcription, DNA-dependent | 1260 | 0.869 | 8 | 1.22E-07 |
| negative regulation of transcription, DNA-dependent | 987 | 0.682 | 7 | 6.69E-07 |
| protein dimerization activity | 996 | 0.688 | 7 | 7.12E-07 |
| protein heterodimerization activity | 374 | 0.258 | 4 | 8.13E-05 |
| protein kinase binding | 376 | 0.26 | 4 | 8.30E-05 |
| kinase binding | 418 | 0.289 | 4 | 0.000125 |
| histone deacetylase binding | 65 | 0.0449 | 2 | 0.000872 |
| steroid hormone receptor binding | 69 | 0.0476 | 2 | 0.000982 |
| chromatin binding | 338 | 0.233 | 3 | 0.00134 |
| protein complex binding | 339 | 0.234 | 3 | 0.00135 |
| identical protein binding | 910 | 0.628 | 4 | 0.0024 |
| nuclear hormone receptor binding | 153 | 0.106 | 2 | 0.00472 |
| protein homodimerization activity | 573 | 0.396 | 3 | 0.006 |
| hormone receptor binding | 176 | 0.122 | 2 | 0.0062 |
| enzyme binding | 1200 | 0.828 | 4 | 0.00654 |
| NF-kappaB binding | 25 | 0.0173 | 1 | 0.0171 |
| regulation of DNA-dependent transcription, elongation | 37 | 0.0256 | 1 | 0.0253 |
| protein deacetylase activity | 39 | 0.0269 | 1 | 0.0266 |
| deacetylase activity | 52 | 0.0359 | 1 | 0.0353 |
| ligand-activated sequence-specific DNA binding RNA polymerase II transcription factor activity | 57 | 0.0394 | 1 | 0.0387 |
| histone methyltransferase activity | 60 | 0.0414 | 1 | 0.0407 |
| chemokine receptor binding | 75 | 0.0518 | 1 | 0.0506 |
| hydrolase activity, acting on carbon-nitrogen (but not peptide) bonds, in linear amides | 76 | 0.0525 | 1 | 0.0513 |
| protein domain specific binding | 560 | 0.387 | 2 | 0.0547 |
| N-methyltransferase activity | 84 | 0.058 | 1 | 0.0565 |
| protein methyltransferase activity | 86 | 0.0594 | 1 | 0.0578 |
| protein N-terminus binding | 86 | 0.0594 | 1 | 0.0578 |
| protein binding transcription factor activity | 600 | 0.414 | 2 | 0.0619 |
| calcium ion binding | 673 | 0.465 | 2 | 0.0758 |
| S-adenosylmethionine-dependent methyltransferase activity | 123 | 0.0849 | 1 | 0.0818 |
| hydrolase activity, acting on carbon-nitrogen (but not peptide) bonds | 147 | 0.102 | 1 | 0.097 |
| transcription corepressor activity | 208 | 0.144 | 1 | 0.135 |
| methyltransferase activity | 222 | 0.153 | 1 | 0.143 |
| G-protein coupled receptor binding | 232 | 0.16 | 1 | 0.149 |
| transferase activity, transferring one-carbon groups | 233 | 0.161 | 1 | 0.15 |
| cytokine receptor binding | 266 | 0.184 | 1 | 0.169 |
| receptor binding | 1590 | 1.1 | 2 | 0.303 |
| transcription cofactor activity | 552 | 0.381 | 1 | 0.322 |
| transport | 4830 | 3.34 | 4 | 0.442 |
| cation binding | 4160 | 2.87 | 3 | 0.582 |
| ATP binding | 1490 | 1.03 | 1 | 0.662 |
| adenyl ribonucleotide binding | 1530 | 1.05 | 1 | 0.672 |
| adenyl nucleotide binding | 1530 | 1.06 | 1 | 0.673 |
| ion binding | 6140 | 4.24 | 4 | 0.677 |
| purine ribonucleotide binding | 1890 | 1.31 | 1 | 0.754 |
| purine nucleotide binding | 1900 | 1.31 | 1 | 0.755 |
| receptor activity | 1920 | 1.33 | 1 | 0.76 |
| zinc ion binding | 2010 | 1.39 | 1 | 0.776 |
| transition metal ion binding | 2270 | 1.57 | 1 | 0.819 |
| nucleotide binding | 2470 | 1.71 | 1 | 0.846 |

**Table S3f. GO Cellular Component enrichement analysis in Shared-DEmiRNA-TF network-interactions**

| **GO-Cellular Component** | **Total** | **Expected** | **Hits** | **P value** |
| --- | --- | --- | --- | --- |
| nuclear lumen | 2690 | 1.61 | 8.00E+00 | 1.51E-05 |
| nucleoplasm | 1820 | 1.09 | 7.00E+00 | 1.65E-05 |
| nuclear part | 3330 | 2 | 8.00E+00 | 7.71E-05 |
| organelle lumen | 3380 | 2.03 | 8.00E+00 | 8.69E-05 |
| nucleoplasm part | 910 | 0.546 | 5.00E+00 | 9.60E-05 |
| membrane-enclosed lumen | 3440 | 2.07 | 8.00E+00 | 9.90E-05 |
| nucleus | 7600 | 4.56 | 1.00E+01 | 0.000389 |
| transcription factor complex | 303 | 0.182 | 3.00E+00 | 0.000649 |
| nuclear matrix | 87 | 0.0522 | 2.00E+00 | 0.00118 |
| protein complex | 4050 | 2.43 | 7.00E+00 | 0.00294 |
| macromolecular complex | 4800 | 2.88 | 7 | 0.00823 |
| histone deacetylase complex | 53 | 0.0318 | 1 | 0.0314 |
| nucleolus | 652 | 0.391 | 2 | 0.0558 |
| basement membrane | 100 | 0.06 | 1 | 0.0584 |
| cytosol | 2660 | 1.6 | 4 | 0.0611 |
| intracellular organelle part | 8620 | 5.17 | 8 | 0.0675 |
| organelle part | 8790 | 5.27 | 8 | 0.0766 |
| nuclear chromatin | 159 | 0.0954 | 1 | 0.0914 |
| sarcomere | 163 | 0.0978 | 1 | 0.0936 |
| contractile fiber part | 187 | 0.112 | 1 | 0.107 |
| myofibril | 197 | 0.118 | 1 | 0.112 |
| extracellular matrix part | 204 | 0.122 | 1 | 0.116 |
| contractile fiber | 214 | 0.128 | 1 | 0.121 |
| spindle | 261 | 0.157 | 1 | 0.146 |
| nuclear chromosome part | 273 | 0.164 | 1 | 0.152 |
| nuclear chromosome | 320 | 0.192 | 1 | 0.176 |
| chromatin | 326 | 0.196 | 1 | 0.179 |
| non-membrane-bounded organelle | 3940 | 2.37 | 4 | 0.193 |
| intracellular non-membrane-bounded organelle | 3940 | 2.37 | 4 | 0.193 |
| proteinaceous extracellular matrix | 398 | 0.239 | 1 | 0.215 |
| actin cytoskeleton | 430 | 0.258 | 1 | 0.23 |
| cytoskeletal part | 1570 | 0.944 | 2 | 0.242 |
| synapse | 558 | 0.335 | 1 | 0.289 |
| extracellular matrix | 570 | 0.342 | 1 | 0.294 |
| chromosomal part | 670 | 0.402 | 1 | 0.337 |
| chromosome | 784 | 0.47 | 1 | 0.382 |
| cytoskeleton | 2200 | 1.32 | 2 | 0.387 |
| microtubule cytoskeleton | 1120 | 0.671 | 1 | 0.501 |
| extracellular region part | 1320 | 0.792 | 1 | 0.562 |
| extracellular region | 2860 | 1.71 | 1 | 0.848 |
| cytoplasmic part | 9740 | 5.84 | 4 | 0.933 |
| plasma membrane | 5500 | 3.3 | 1 | 0.982 |
| cytoplasm | 13100 | 7.88 | 5 | 0.991 |
| membrane | 11700 | 7.03 | 1 | 1 |

**Table S4. shared-DEmiRNA-compound network-interactions**

| **Node** | **Degree** | **Betweenness** |
| --- | --- | --- |
| hsa-mir-155 | 4 | 58.08333 |
| 5-fluorouracil | 4 | 27.41667 |
| Curcumin | 4 | 33.41667 |
| 1,2,6-Tri-O-galloyl-beta-D-glucopyranose | 4 | 22.33333 |
| hsa-mir-30a | 3 | 15.25 |
| hsa-mir-143 | 3 | 16.5 |
| Glucocorticoid | 3 | 14.41667 |
| hsa-mir-223 | 2 | 6 |
| Cisplatin | 2 | 3.416667 |
| Doxorubicin | 2 | 39 |
| Formaldehyde | 2 | 15 |
| hsa-mir-652 | 2 | 28 |
| hsa-mir-204 | 2 | 5.583333 |
| hsa-mir-29a | 2 | 1.583333 |
| hsa-mir-650 | 1 | 0 |
| hsa-mir-95-5p | 1 | 0 |
| hsa-mir-484 | 1 | 0 |
